# Reprogramming the tumor microenvironment leverages CD8^+^ T cell responses to a shared tumor/self antigen in ovarian cancer

**DOI:** 10.1016/j.omto.2023.02.002

**Published:** 2023-02-09

**Authors:** Anna Mistarz, Marta Winkler, Sebastiano Battaglia, Song Liu, Alan Hutson, Hanna Rokita, Andrea Gambotto, Kunle O. Odunsi, Prashant K. Singh, A.J. Robert McGray, Jianmin Wang, Danuta Kozbor

**Affiliations:** 1 Department of Immunology, Roswell Park Comprehensive Cancer Center, Buffalo, NY 14263, USA; 2 Department of Biostatistics and Bioinformatics, Roswell Park Comprehensive Cancer Center, Buffalo, NY 14263, USA; 3 Faculty of Biochemistry, Biophysics, and Biotechnology, Jagiellonian University, 30-387 Kraków, Poland; 4 Department of Surgery, University of Pittsburgh, Pittsburgh, PA 15261, USA; 5 University of Chicago Comprehensive Cancer Center, Chicago, IL 60637, USA; 6 Cancer Genetics and Genomics, Roswell Park Comprehensive Cancer Center, Buffalo, NY 14263, USA

**Keywords:** immunotherapy, ovarian cancer, tolerance, tumor microenvironment, CXCR4 antagonist, oncolityc vaccinia virus

## Abstract

Tumor antigen-driven responses to weakly immunogenic self-antigens and neoantigens directly affect treatment efficacy following immunotherapy. Using orthotopically grown SV40 T antigen^+^ ovarian carcinoma in antigen-naive wild-type or Tg*MISIIR-TAg-Low* transgenic mice expressing SV40 T antigen as a self-antigen, we investigated the impact of CXCR4-antagonist-armed oncolytic virotherapy on tumor progression and antitumor immunity. Immunostaining and single-cell RNA sequencing analyses of the peritoneal tumor microenvironment of untreated tumors in syngeneic wild-type mice revealed the presence of SV40 T antigen-specific CD8^+^ T cells, a balanced M1/M2 transcriptomic signature of tumor-associated macrophages, and immunostimulatory cancer-associated fibroblasts. This contrasted with polarized M2 tumor-associated macrophages, immunosuppressive cancer-associated fibroblasts, and poor immune activation in Tg*MISIIR-TAg-Low* mice. Intraperitoneal delivery of CXCR4-antagonist-armed oncolytic vaccinia virus led to nearly complete depletion of cancer-associated fibroblasts, M1 polarization of macrophages, and generation of SV40 T antigen-specific CD8^+^ T cells in transgenic mice. Cell depletion studies revealed that the therapeutic effect of armed oncolytic virotherapy was dependent primarily on CD8^+^ cells. These results demonstrate that targeting the interaction between immunosuppressive cancer-associated fibroblasts and macrophages in the tolerogenic tumor microenvironment by CXCR4-A-armed oncolytic virotherapy induces tumor/self-specific CD8^+^ T cell responses and consequently increases therapeutic efficacy in an immunocompetent ovarian cancer model.

## Graphical abstract

**Figure:**
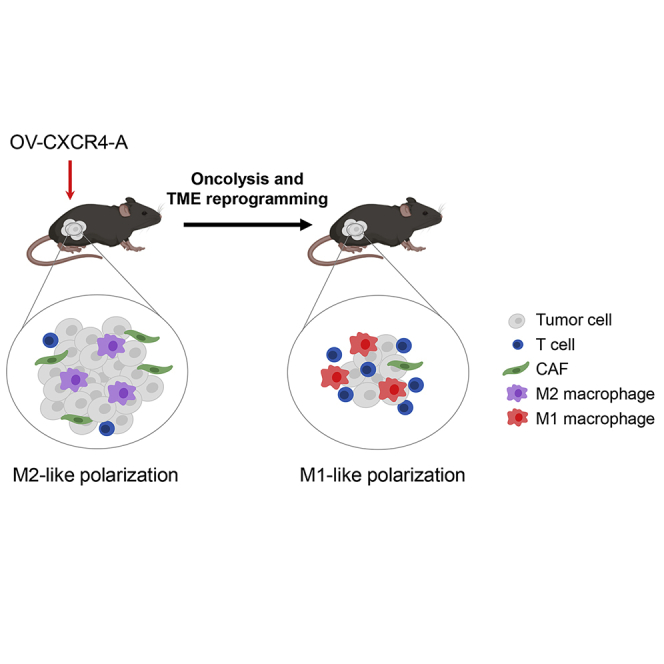

## Teaser

Induction of immunity against self and neoantigens is a prerequisite for successful cancer immunotherapy. Targeting the interaction between immunosuppressive cancer-associated fibroblasts and macrophages in the tolerogenic tumor microenvironment by CXCR4-A-armed oncolytic virotherapy transiently reverses CD8^+^ T cell tolerance to tumor/self-antigen and increases therapeutic efficacy in an immunocompetent ovarian cancer model.

## Introduction

Therapeutic strategies that can generate immunity against self and neoantigens are considered a prerequisite for successful cancer immunotherapy. Although tumor antigens that are self-proteins are often weakly immunogenic because of pre-existing tolerance,^1^^,^^2^ growing evidence demonstrates that immune tolerance to self can be overcome with robust stimulation with self-antigens in the context of infection, resulting in targeted expansion of tumor/self-specific T cells that participate in tumor control without induction of serious toxicities.^3^^,^^4^^,^^5^ In this regard, therapeutic strategies focusing on *in situ* vaccination by oncolytic virotherapy show promise because oncolytic viruses are designed to generate immune responses to tumor antigens during oncolysis of cancer cells by induction of immunogenic cell death,^6^ leading to increased type 1 interferon production.^7^ In line with these observations, advanced melanoma patients treated with an engineered herpes virus developed tumor-specific (MART-1) T cells within injected and non-injected lesions,^8^ suggesting that virotherapy-mediated epitope spreading has the potential to serve as personalized immunotherapy. We have recently demonstrated that the innate resistance of metastatic ovarian tumors in syngeneic mice could be overcome by an oncolytic vaccinia virus-delivered CXCR4 antagonist (OV-CXCR4-A)^9^ by reducing tumor load and the immunosuppressive network, leading to intratumoral accumulation of CD8^+^ T cells and increased overall survival compared with tumor-bearing mice treated with control viruses.^10^^,^^11^^,^^12^ Because the CXCR4/CXCL12 axis plays multiple pleiotropic roles in the progression of ovarian cancer, including stimulation of angiogenesis,^13^ immune suppression by the fibroblast activation protein (FAP)^+^ cancer-associated fibroblasts (CAFs),^14^ recruitment of endothelial progenitor cells,^15^ as well as accumulation of CD11b^+^Gr1^+^ myeloid-derived suppressor cells (MDSCs)^16^ and regulatory T (Treg) cells,^17^ modulation of this axis impacts immune mechanisms of tumor destruction.^18^ However, how immune and nonimmune cell subsets contribute to tolerance remains incompletely understood. Similarly, despite numerous studies showing that CXCR4 antagonists demonstrated antitumor efficacy in syngeneic murine models and have been evaluated in early clinical trials,^19^^,^^20^^,^^21^ there is a limited understanding of how generation of antitumor T cell responses to tumor/self-antigen by OV-CXCR4-A is affected by the tolerogenic tumor microenvironment (TME).

Here, we compared the antitumor activity of CD8^+^ T cells against SV40 T antigen (TAg)^+^ murine ovarian carcinoma (MOVCAR) 5009 ovarian carcinoma (OC) grown orthotopically in TAg-naive syngeneic wild-type (WT) C57BL/6 mice to responses generated in non-tumor-prone C57BL/6 Tg*MISIIR-TAg-Low* transgenic mice expressing the TAg protein in epithelial cells lining the fallopian tubes under transcriptional control of the Müllerian inhibiting substance type II receptor gene promoter.^22^ To generate C57BL/6 Tg*MISIIR-TAg-Low* mice, TAg-positive male founders were bred to WT mice to produce female offspring exhibiting low TAg transgene expression in the fallopian tubes without obvious pathology.^23^ Independent MOVCAR cell lines recapitulating essential features of serous OC were established from ascites of C57BL/6 Tg*MISIIR-TAg* mice expressing a high level of TAg.^22^ One of these cell lines, MOVCAR 5009, was selected based on tumorigenicity in WT and Tg*MISIIR-TAg-Low* transgenic mice.^22^^,^^24^ The availability of these syngeneic WT and Tg*MISIIR-TAg-Low* mice allowed us to compare the *in vivo* effects of TAg expressed as a neoantigen and tumor/self-antigen, respectively, on generation of TAg-specific CD8^+^ T cells and OV-CXCR4-A treatment efficacy. Our results showed that transient antigen-specific reversal of CD8^+^ T cell tolerance to TAg in tumor-bearing Tg*MISIIR-TAg-Low* mice was achieved by OV-CXCR4-A-induced immunogenic cell death and transcriptional reprogramming of M2 tumor-associated macrophages (TAMs) associated with effective targeting of immunosuppressive CAFs by the armed virus.

## Results

### Reduced antitumor efficacy of OV-CXCR4-A in Tg*MISIIR-TAg-Low* mice compared with their WT counterparts

**Figure 1:**
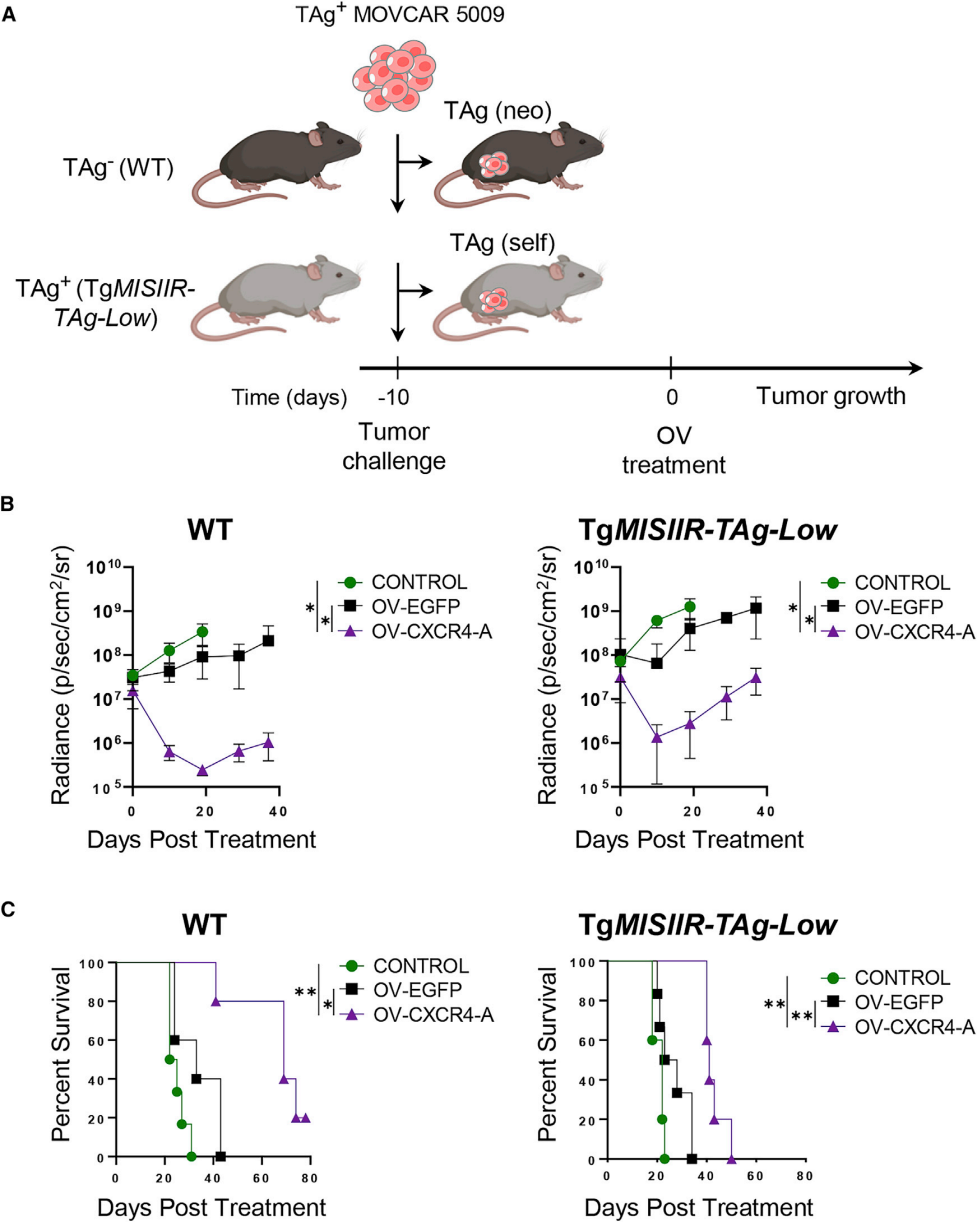
The effect of OV-CXCR4-A treatment on progression of MOVCAR 5009 tumor growth in WT and Tg*MISIIR-TAg-Low* mice (A) An experimental scheme. Syngeneic WT or Tg*MISIIR-TAg-Low* mice (n = 5–6 per group) were injected i.p. with 5 × 10^6^ MOVCAR 5009 cells 10 days before treatment with OV-EGFP or OV-CXCR4-A. Control mice were treated with PBS. (B) Tumor volume curves in WT (left panel) and Tg*MISIIR-TAg-Low* (right panel) mice after different treatments. Individual data points represent mean ± SD. ∗p < 0.05. (C) Survival was defined as the point when mice were euthanized because of development of abdominal distention. Kaplan-Meier survival plots were prepared, and significance was calculated using the log rank method. ∗p < 0.05, ∗∗p < 0.01.

To analyze the effect of tumor/self-antigen expression in the host on the efficacy of OV-CXCR4-A treatment, we compared the progression of TAg^+^ MOVCAR 5009 tumors in syngeneic WT and Tg*MISIIR-TAg-Low* mice (Figure 1A). For the analysis, MOVCAR 5009 cells (5 × 10^6^/mouse) were injected intraperitoneally (i.p.) into both groups of mice (n = 5–6 mice/group) and treated intratumorally with 5 × 10^7^ plaque-forming units (PFUs)/mouse of a green fluorescence protein-expressing virus (OV-EGFP) or OV-CXCR4-A, while control mice received PBS. Kinetics analysis of tumor growth, quantified by bioluminescence imaging (Figure S1), revealed that OV-CXCR4-A exhibited significant therapeutic efficacy compared with the control OV-EGFP virus in WT and Tg*MISIIR-TAg-Low* mice (Figure 1B; p = 0.02 and p = 0.04, respectively). Although OV-EGFP treatment reduced tumor progression and increased survival compared with the untreated tumors in WT and transgenic mice (Figures 1B and 1C), the differences did not reach statistically significant levels, consistent with our previous studies using oncolytic virotherapy to target murine ID8-T and human CAOV2 ovarian cancer cells in syngeneic and xenograft murine models, respectively.^10^ Further, we observed that the rate of tumor growth after OV-CXCR4-A treatment was significantly slower in WT compared with Tg*MISIIR-TAg-Low* mice (Figure 1B; p = 0.02), which was reflected by longer survival periods (Figure 1C; median survival 69 days versus 41 days, respectively). Given that identical cancer cells were injected into all animals, these results indicated that the microenvironments diverged quickly between WT and Tg*MISIIR-TAg-Low* mice and impacted the ability to generate antitumor immunity after OV-CXCR4-A treatment through immune modulation.

### MOVCAR 5009-challenged Tg*MISIIR-TAg-Low* mice are enriched in immunosuppressive subsets of myeloid cells

**Figure 2:**
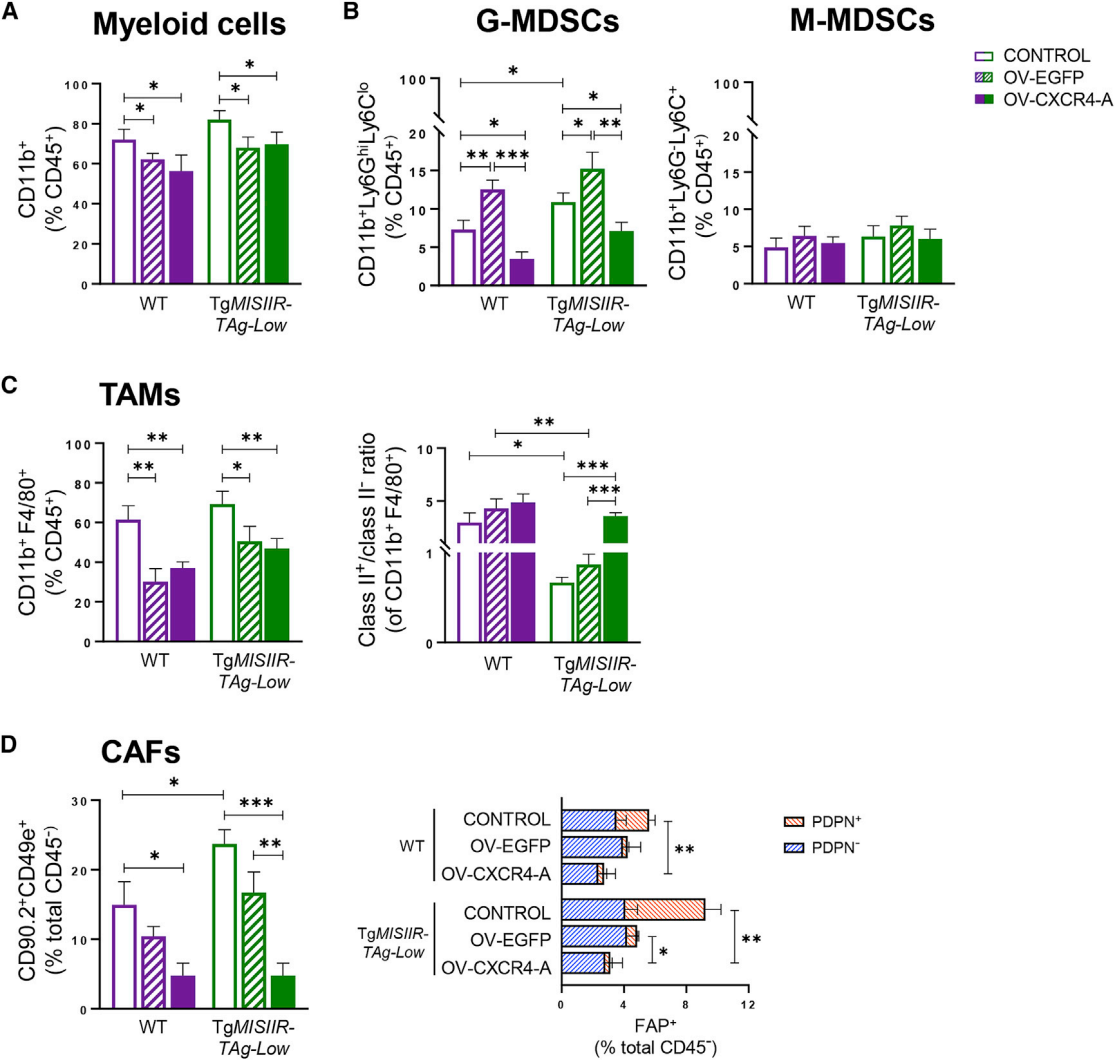
The effect of OV-EGFP and OV-CXCR4-A on accumulation of peritoneal myeloid cells and CAFs in MOVCAR 5009 tumor-bearing WT and Tg*MISIIR-TAg-Low* mice (A and B) Single-cell suspensions isolated from the peritoneal fluid of tumor-bearing mice were analyzed by flow cytometry for the percentages of (A) CD11b^+^ myeloid cells and (B) CD11b^+^Ly6G^hi^Ly6C^lo^ G-MDSCs (left panel) and CD11b^+^Ly6G^−^Ly6C^hi^ M-MDSCs (right panel) among CD45^+^ leukocytes. ∗p < 0.05, ∗∗p < 0.01, ∗∗∗p < 0.001. (C) The left panel depicts the percentages of CD11b^+^F4/80^+^ TAMs among CD45^+^ leukocytes, and the right panel shows the ratios of class II^+^ to class II^−^ TAMs in WT and Tg*MISIIR-TAg-Low* mice. Data points represent the mean of 5 mice per group ± SD. ∗p < 0.05, ∗∗p < 0.01, ∗∗∗p < 0.001. (D) Relative proportions of tumor-infiltrating CD90.2^+^CD49e^+^ CAFs are presented as a percentage of total CD45^−^ cells (left panel). FAP^+^ CAFs were depicted as PDPN^+^ or PDPN^−^ (right panel). Results are presented as mean ± SD. ∗p < 0.05, ∗∗p < 0.01, ∗∗∗p < 0.001.

Based on our previous studies demonstrating that enhanced inhibition of tumor growth following OV-CXCR4-A treatment was associated with induction of antitumor immune responses,^9^^,^^10^^,^^11^^,^^12^ we compared the potential of OV-EGFP and OV-CXCR4-A to modulate the peritoneal TME and promote effective antigen presentation, leading to loss of immune tolerance to tumor/self-antigens. Flow cytometry analyses of single-cell suspensions isolated from peritoneal fluids of untreated and virotherapy-treated mice 10 days after OV-EGFP or OV-CXCR4-A administration revealed robust accumulation of CD11b^+^ myeloid cells in control tumors (>70% of CD45^+^ leukocytes; Figure 2A), with reduced numbers after virotherapy treatment in WT and Tg*MISIIR-TAg-Low* mice (p < 0.05). Among the CD11b^+^ myeloid populations, the frequency of Ly6G^high^Ly6C^low^ granulocytic MDSCs (G-MDSCs) was significantly lower in WT mice than those in their tumor-bearing transgenic counterparts (Figure 2B, left panel; p = 0.02). Treatment with OV-EGFP increased the percentage of G-MDSCs compared with the untreated controls in both groups of mice (Figure 2B; p < 0.05). In contrast, the accumulation of G-MDSCs in OV-CXCR4-A-treated tumors was significantly reduced compared with their untreated and OV-EGFP-treated counterparts in WT mice (Figure 2B; p = 0.02 and p = 0.0005, respectively) and Tg*MISIIR-TAg-Low* mice (Figure 2B; p = 0.02 and p = 0.005, respectively). No changes between treatment groups were detected for CD11b^+^Ly6G^−^Ly6C^+^ monocytic MDSCs (M-MDSCs), which in all cases comprised less than 10% of the CD45^+^ leukocytes (Figure 2B, right panel).

Because CD11b^+^ F4/80^+^ macrophages can control the dissemination of ovarian tumors in the peritoneal cavity and influence the efficacy of antitumor therapy, ^25^ we used flow cytometry to examine their accumulation in the peritoneal TME (referred to as TAMs) in untreated and virotherapy-treated WT and Tg*MISIIR-TAg-Low* mice (Figure 2C, left panel, and S2). The frequency of CD11b^+^F4/80^+^ TAMs in the CD45^+^ leukocyte population decreased after OV-EGFP and OV-CXCR4-A treatment compared with untreated WT mice (p = 0.004 and p = 0.005, respectively) and transgenic mice (p = 0.03 and p = 0.009, respectively). We also observed pronounced differences with regard to major histocompatibility complex (MHC) class II expression on TAMs, a lack of which is associated with their immunosuppressive phenotype,^26^ between untreated tumor-bearing WT and transgenic mice (Figures S2 and 2C, right panel). The ratios of MHC class II^+^ to MHC class II^−^ CD11b^+^F4/80^+^ TAMs in the untreated tumors of WT mice ranged between 2.0 and 3.8 (mean 2.9 ± 0.9) and exhibited small increases after OV-EGFP and OV-CXCR4-A treatments (mean 4.3 ± 0.9 and 4.9 ± 0.8, respectively). In contrast, the ratios of MHC class II^+^/MHC class II^−^ TAMs were reversed in untreated and OV-EGFP-treated tumors of Tg*MISIIR-TAg-Low* mice (mean 0.7 ± 0.06 and 0.8 ± 0.11, respectively), and significantly lower compared with their counterparts in WT tumors (p = 0.01 and p = 0.003, respectively). After OV-CXCR4-A treatment of the tolerogenic TME in Tg*MISIIR-TAg-Low* mice, the ratios of MHC class II^+^/MHC class II^−^ TAMs (mean 3.6 ± 0.3) were similar to those measured in OV-CXCR4-A-treated WT tumors and significantly higher compared with untreated and OV-EGFP-treated tumors (Figure 2C, right panel; p = 0.0001 and 0.0002, respectively).

### Accumulation of CAFs in the ovarian TME is reduced by OV-CXCR4-A treatment

Because CAFs constitute a major population within the stroma of all solid tumors, including OC, in which they often exert protumorigenic functions,^27^ we next examined whether the qualitative differences within TAM subsets between tumors of WT and Tg*MISIIR-TAg-Low* mice also reflect changes in the CAFs subsets.^28^ Phenotypic analysis of single-cell suspensions isolated from the peritoneal fluid of MOVCAR 5009-bearing mice using the pan-CAF markers CD49e and CD90.2 along with linage markers to exclude hematopoietic (CD45) and endothelial (CD31) cells (Figures S3A and S3B) revealed higher frequencies of CAFs in untreated MOVCAR 5009 tumors in Tg*MISIIR-TAg-Low* than WT mice (Figure 2D, left panel; p = 0.02). This was largely attributed to the accumulation of CAFs expressing both FAP and podoplanin (PDPN) (Figure 2D, right panel), reported previously to restrain the proliferation of activated T cells in a nitric oxide-dependent manner.^29^ Consistent with previous findings that proliferating CAFs exhibit increased sensitivity to oncolytic viruses compared with normal fibroblasts,^30^^,^^31^^,^^32^ the numbers of CAFs decreased after OV-EGFP treatment compared with untreated tumors, although the differences did not reach significance (Figure 2D, left panel). The impact of OV on reducing i.p. CAFs was magnified by OV-CXCR4-A treatment, resulting in a significant decrease in the percentage of CAFs compared with untreated tumors in WT and transgenic mice (Figure 2D, left panel; p = 0.014 and p = 0.0003, respectively) and OV-EGFP-treated transgenic mice (p = 0.004). Of note, the OV-CXCR4-A treatment virtually ablated the FAP^+^PDPN^+^ CAF subset in both groups of mice (Figure 2D, right panel; p < 0.01).

### OV-CXCR4-A treatment induces antigen-specific CD8^+^ T cell responses in MOVCAR 5009-challenged Tg*MISIIR-TAg-Low* mice

**Figure 3:**
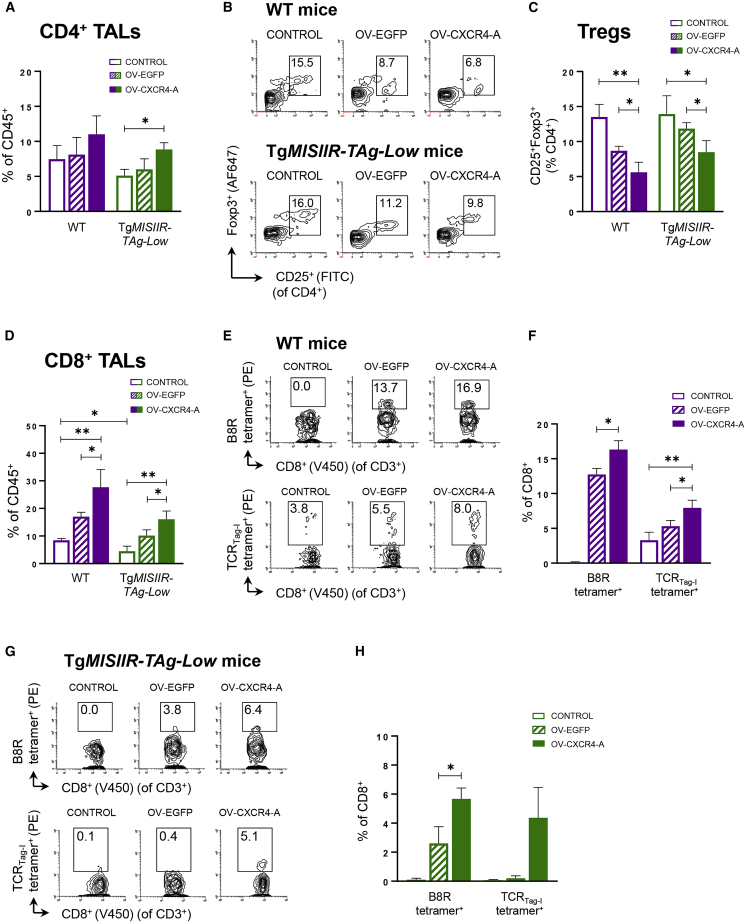
The changes in i.p. accumulation of CD4^+^ and CD8^+^ TALs and their antigenic specificity after OV-EGFP and OV-CXCR4-A treatment of MOVCAR 5009 tumors in WT and Tg*MISIIR-TAg-Low* mice (A) The percentages of CD4^+^ TALs of total CD45^+^ leukocytes in the peritoneal TME of untreated and OV-EGFP- and OV-CXCR4-A-treated mice were analyzed 10 days after the viral challenge and presented as mean ± SD. ∗p < 0.05. (B and C) Flow cytometry plots demonstrating the accumulation of CD4^+^CD25^+^Foxp3^+^ T lymphocytes in peritoneal TME of untreated and OV (OV-EGFP and OV-CXCR4-A)-treated WT and transgenic mice (B) and percentages of Treg cells of CD4^+^ TALs (C) (n = 5). ∗p < 0.05, ∗∗p < 0.01. (D) The differences in percentages of CD8^+^ TALs between different groups of mice and treatments were analyzed and presented as mean ± SD. ∗p < 0.05, ∗∗p < 0.01. (E and F) Representative flow cytometry plots demonstrating specific binding of B8R tetramer^+^ and TCR_Tag-I_ tetramer^+^ to CD8^+^ TALs (E, top and bottom panels, respectively) and percentages of tetramer^+^ CD8^+^ TALs among total CD45^+^ leukocytes in the peritoneal TME of WT mice (F). ∗p < 0.05, ∗∗p < 0.01. (G and H) Flow cytometry plots demonstrating specific binding of B8R tetramer^+^ and TCR_Tag-I_ tetramer^+^ to CD8^+^ TALs (G, top and bottom panels, respectively) and percentages of tetramer-positive CD8^+^ TALs among total CD45^+^ leukocytes in the peritoneal TME of Tg*MISIIR-TAg-Low* mice (H) (n = 3–5). ∗p < 0.05. (C, F , and H) Data are presented as mean ± SD.

Prompted by the findings that inhibition of the interaction of CXCL12 with CXCR4 on CAFs leads to elimination of tumor cells through accumulation of cytotoxic CD8^+^ T cells,^14^ we next examined the effect of OV-CXCR4-A treatment on tumor-associated lymphocytes (TALs) in the peritoneal TME of WT and transgenic mice. Flow cytometry analyses revealed that the peritoneal TMEs of untreated mice were poorly infiltrated by CD4^+^ TALs that comprised approximately 10% of CD45^+^ leukocytes (Figure 3A). There were small increases in the percentages of CD4^+^ TALs in OV-EGFP- and OV-CXCR4-A-treated tumors within each group of mice compared with their respective controls, with a significant difference detected only after OV-CXCR4-A treatment in the transgenic mice (p = 0.01). Approximately 15% of CD4^+^ T cells in the untreated peritoneal TMEs expressed CD25 and Foxp3 antigens, characteristic of Treg cells (Figures 3B and 3C). In concordance with the findings that CXCL12 induces intratumoral localization of CD4^+^CD25^+^Foxp3^+^ Treg cells in OC,^33^ inhibition of the CXCL12/CXCR4 signaling pathway by OV-CXCR4-A resulted in significantly lower percentages of Treg cells infiltrating the peritoneal TME compared with untreated and OV-EGFP-treated tumors in WT mice (Figure 3C; p = 0.004 and p = 0.03, respectively). Similarly, Treg cell frequencies in the peritoneal cavity of Tg*MISIIR-TAg-Low* mice decreased after OV-CXCR4-A treatment compared with untreated and OV-EGFP-treated counterparts (Figure 3C; p = 0.03 and p = 0.04), although differences between the respective treatment groups in WT and Tg*MISIIR-TAg-Low* mice did not reach significance.

The i.p. accumulation of CD8^+^ TALs after OV-CXCR4-A treatment in WT mice was increased compared with their CD4^+^ counterparts and comprised over 20% of CD45^+^ leukocytes, which was significantly elevated compared with untreated and OV-EGFP-treated tumors (Figure 3D; p = 0.007 and p = 0.049, respectively). The percentages of CD8^+^ TALs in untreated Tg*MISIIR-TAg-Low* mice were lower than those in tumor-bearing WT mice (Figure 3D, p = 0.03), but their numbers increased after OV-CXCR4-A treatment compared with untreated and OV-EGFP-treated tumors (Figure 3D; p = 0.005 and p = 0.04). Based on these data demonstrating increased accumulation of CD8^+^ TALs, we next examined the effect of the peritoneal TME on induction of antigen-specific CD8^+^ T cell responses to the vaccinia virus B8R protein and the SV40 TAg antigen by staining with B8R-K^b^/TSYKFESV and TAg-D^b^/SAINNYAQKL (TCR_Tag-I_) tetramers, respectively. As shown in Figures 3E, top panel, and 3F, approximately 13% of CD8^+^ TALs in WT mice were B8R specific following treatment with OV-EGFP, and this was further increased in OV-CXCR4-A-treated mice (Figure 3F; p = 0.02), presumably because of decreases in immunosuppressive elements in the TME after the armed virotherapy treatment. As expected, no vaccinia-specific responses were detected in untreated mice. Over 3% of CD8^+^ TALs in control tumors of WT mice were Tag specific, and their percentages increased after OV-EGFP treatment, although the differences were not significant (Figures 3E, bottom panel, and 3F). The OV-CXCR4-A treatment was most effective in generating TAg-specific CD8^+^ T cell responses with significant increases in TCR_Tag-I_^+^CD8^+^ TALs compared with untreated or OV-EGFP-treated tumors (Figure 3F; p = 0.007 and p = 0.03). The same analysis performed in Tg*MISIIR-TAg-Low* mice revealed that the levels of B8R-specific T cell responses generated by OV-EGFP or OV-CXCR4-A were over 2-fold lower compared with those detected in their WT counterparts and did not exceed 7% of CD8^+^ TALs (Figures 3G, top panel, and 3H). Similar to WT mice, OV-CXCR4-A generated higher numbers of CD8^+^ B8R-K^b^/TSYKFESV tetramer^+^ TALs compared with those detected in OV-EGFP-treated tumors (p = 0.02). On the other hand, TCR_Tag-I_^+^CD8^+^ TALs were not detectable in untreated or OV-EGFP-treated tumors of Tg*MISIIR-TAg-Low* mice, and TAg-specific CD8^+^ T cell responses were generated only in OV-CXCR4-A-treated transgenic tumors (Figures 3G, bottom panel, and 3H). Furthermore, the TCR_Tag-I_ tetramer binding to CD8^+^ TALs was approximately 4-fold higher in WT mice compared with Tg*MISIIR-TAg-Low* mice (Figures 3E and 3G, bottom panels; mean fluorescence intensity [MFI] = 93.3 ± 7.5 versus MFI = 22.3 ± 2.5, p = 0.001), which could reflect differences in activation state reported previously for tolerant self-specific T cells to ovalbumin in intestinal fatty acid-binding protein promoter-ovalbumin (iFABP-Ova) mice.^3^

### Depletion of CD8^+^ T cells abrogates the therapeutic efficacy of OV-CXCR4-A treatment

**Figure 4:**
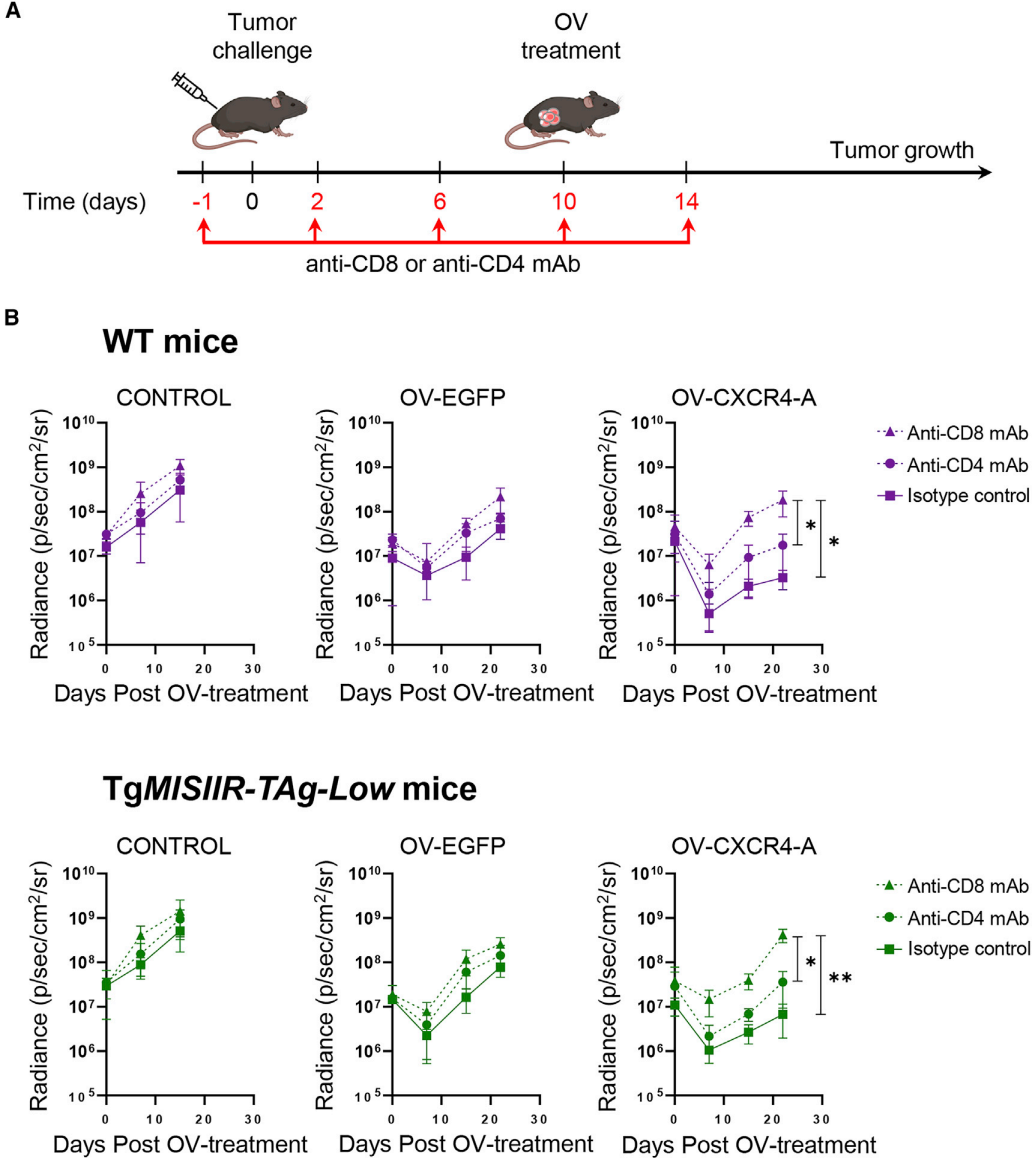
CD4 and CD8 T cell depletion in WT and Tg*MISIIR-TAg-Low* mice (A) An experimental scheme. WT or Tg*MISIIR-TAg-Low* mice (n = 5/group) were injected i.p. with 5 × 10^6^ MOVCAR 5009 cells 10 days before treatment with OV-EGFP or OV-CXCR4-A. Control mice were treated with PBS. Anti-CD8 or anti-CD4 Abs (100 μg/injection) were injected i.p. 1 day before and on days 2, 6, 10, and 14 after the tumor challenge. (B) Tumor volume curves in WT (top panel) and Tg*MISIIR-TAg-Low* (bottom panel) mice after different treatments. Individual data points represent mean ± SD. ∗p < 0.05; ∗∗p<0.01.

To determine the contribution of CD4^+^ and CD8^+^ T cell responses to the therapeutic benefit of OV-CXCR4-A treatment, we performed depletion studies by treating mice with anti-CD4 (clone YTS 191) or anti-CD8 (clone YTS 169.4) monoclonal antibodies (mAbs) by i.p. injection before and after tumor challenge Figure 4A).^34^ Treatment with an anti-keyhole limpet hemocyanin antibody (clone LTF-2) served as an isotype control.^34^ MOVCAR 5009 cells were injected i.p. 1 day after initiation of T cell depletion, and mice were treated with OV-EGFP or OV-CXCR4-A 10 days later. As shown in Figure 4B, top panel, CD8 depletion in MOVCAR 5009 tumor-bearing WT mice abrogated OV-CXCR4-A treatment-mediated control of tumor growth (p = 0.03), which was more pronounced compared with depletion of CD4^+^ T cells. On the other hand, the effects of CD8 depletion in untreated or OV-EGFP-treated WT mice were modest and did not reach significant levels compared with their respective isotype-treated controls, although small but transient inhibition of tumor growth within the first 10 days of OV-EGFP treatment was observed, likely because of the cytolytic effect of the virus on tumor cells regardless of T cell depletion. Depletion of CD4^+^ or CD8^+^ T cells in untreated and OV-EGFP-treated tumor-bearing Tg*MISIIR-TAg-Low* mice had practically no antitumor effects (Figure 4B, bottom panel), consistent with the absence of TAg-specific TALs in peritoneal cavities in these mice. In contrast, depletion of CD8^+^ T cells in transgenic mice abrogated the OV-CXCR4 treatment-mediated control of tumor growth, restoring primary tumor growth to the level of untreated mice (p = 0.007). Cumulatively, these data showed that the reduced growth rate of MOVCAR 5009 tumors in OV-CXCR4-A-treated mice was dependent on therapy-induced CD8^+^ T cell responses. Furthermore, the effect of OV-EGFP alone was modest, produced only intermediate changes in the TME, and was insufficient to break self-tolerance. In contrast, the improved efficacy of OV-CXCR4-A was associated with robust immune changes and induction of TAg-specific CD8^+^ TALs.

### OV-CXCR4-A treatment overcomes the immunosuppressive landscape of TAMs in tumor-bearing Tg*MISIIR-TAg-Low* mice

**Figure 5:**
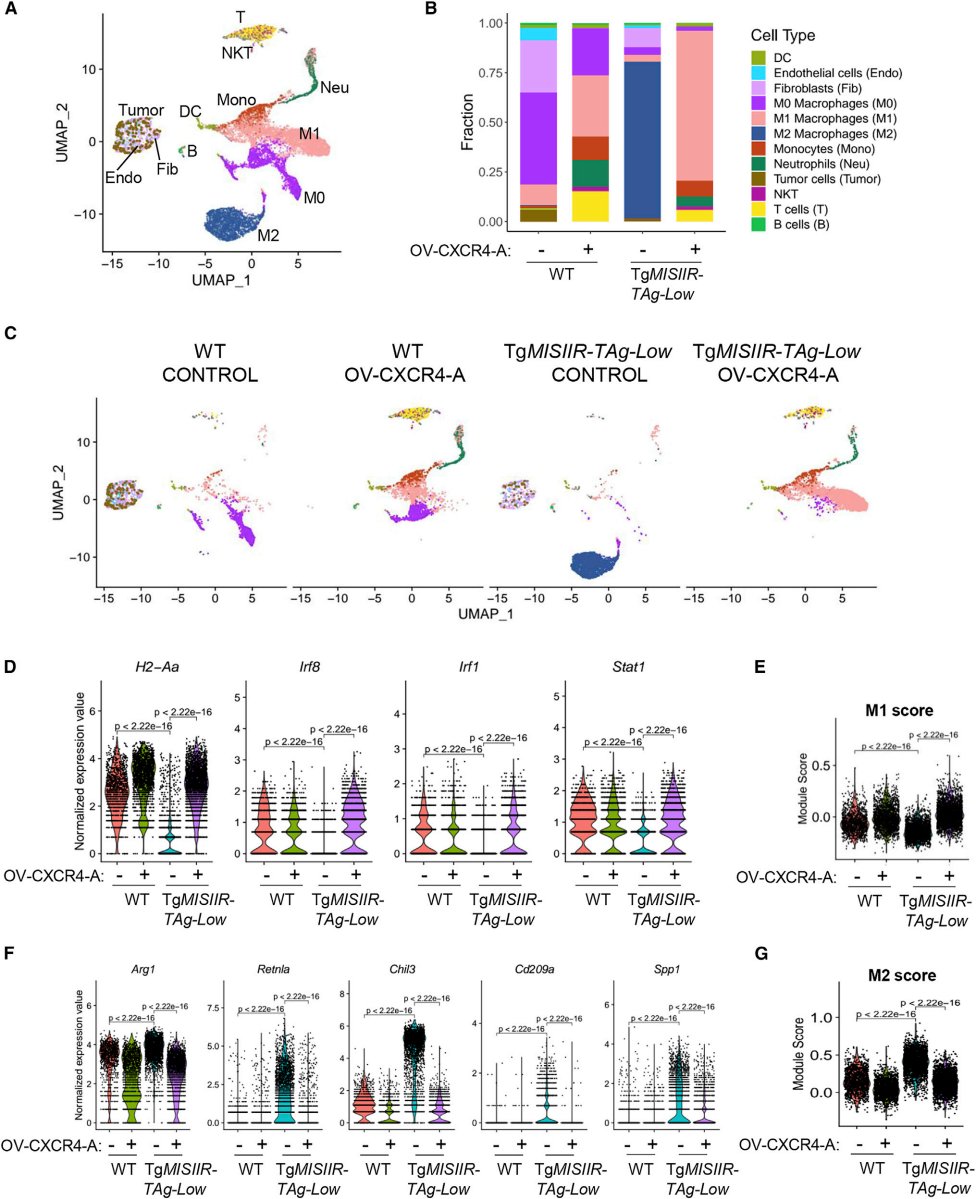
OV-CXCR4-A-mediated changes in the immune profile of myeloid populations in MOVCAR 5009-bearing mice (A and B) Uniform manifold approximation and projection (UMAP) plots of the peritoneal TME in untreated and OV-CXCR4-A-treated MOVCAR 5009 tumors were analyzed 10 days after the treatment. Unsupervised clustering analysis followed by cell type annotation and differential expression analyses revealed the accumulation of different subsets of TAMs (M0, M1, and M2), neutrophils, DCs, T cells/NK T cells, fibroblasts, endothelial cells, and tumor cells in individual clusters. (C) The fraction graph shows the distribution of cell type populations in control and OV-CXCR4-A-treated tumors in WT and Tg*MISIIR-TAg-Low* mice. Cell types on UMAP plots are highlighted by matching colors in the data panel. (D) Violin plots showing expression of selected M1 genes (*H2-Aa*, *Irf8*, *Irf1*, and *Stat1*) in TAMs with significant changes in gene expression. (E) M1 scores with significant changes in gene expression between the indicated groups. (F) Violin plots showing expression of selected M2 genes (*Arg1*, *Retnla*, *Chil3*, *Cd209a*, and *Spp1*) in TAMs with significant changes in gene expression. (G) M2 scores with significant changes in gene expression between the indicated groups.

To better delineate local changes that permitted effective braking of immune tolerance, we performed a global analysis of the cellular landscape of the peritoneal TME by single-cell RNA sequencing (scRNA-seq) profiling during induction of TAg-specific CD8^+^ TALs by OV-CXCR4-A. The scRNA-seq profiling of the TME, used for exploring differences in the remodeling of different compartments in the peritoneum in WT and Tg*MISIIR-TAg-Low* mice before and after OV-CXCR4-A treatment, confirmed the myeloid-driven phenotype of MOVCAR 5009 tumors in both groups of mice (Figures 5A and 5B). The clustering analysis presented in Figure S4 with cell type annotation (Figure 5A) and the fraction graph (Figure 5B) revealed that the majority of monocytes/macrophages were organized in cluster 0 (C0; M2 TAMs), C1, C6 (M1 TAMs), C2 (monocytes/M1 TAMs), C4, C7, and C9 (M0 TAMs). Other clusters contained neutrophils (C6 and C8), DCs (C10), B cells (C12), and T cells/natural killer (NK) T cells (C5 and C11), whereas CD45^−^ fibroblasts and endothelial and tumor cells were organized together in C3. Consistent with the highly heterogeneous transcriptomic profile of individual clusters (Figure S5), cells isolated from the peritoneal TME of untreated and OV-CXCR4-A-treated mice demonstrated differential cluster distribution based on a WT versus Tg*MISIIR-TAg-Low* mouse background (Figure 5C). For example, the lymphoid clusters (T cells/NK T cells) were prominent in OV-CXCR4-A-treated tumors but underrepresented in control tumor-bearing mice, opposite to the cluster depicting fibroblasts, endothelial, and tumor cells prevailing in untreated tumors, whereas the M2 macrophage cluster was present only in the untreated group of Tg*MISIIR-TAg-Low* mice. The heterogeneity within the TAM subsets also paralleled different profiles of antigen expression that are known to be associated with M1 and M2 anti- and pro-tumorigenic activities, respectively. Analyses of signature genes associated with M1 (Figures 5D and 5E) and M2 phenotypes (Figures 5F and 5G) revealed a lower expression of M1 markers, which are essential for generation of type 1 responses (*H2-Aa*, *Irf8*, *Irf1*, and *Stat1*) in the control tumors of Tg*MISIIR-TAg-Low* mice compared with their WT counterparts. This profile of gene expression in the tolerogenic TME was concomitant with the upregulation of key markers of M2 macrophages and MDSCs that mediate T cell suppression (*Arg1*, *Retnla*, and *Chil3*),^35^^,^^36^^,^^37^ promote CD4^+^Foxp3^+^ Treg cell expansion (*Cd209a*),^38^ as well as foster angiogenesis and modify tumor-associated inflammatory cell migration and function (*Spp1*).^39^^,^^40^ This transcriptomic M2 signature detected in the control tumors of Tg*MISIIR-TAg-Low* mice was no longer observed following OV-CXCR4-A treatment, resulting in similar M1-to-M2 scores in both groups of mice (Figures 5E and 5G).

**Figure 6:**
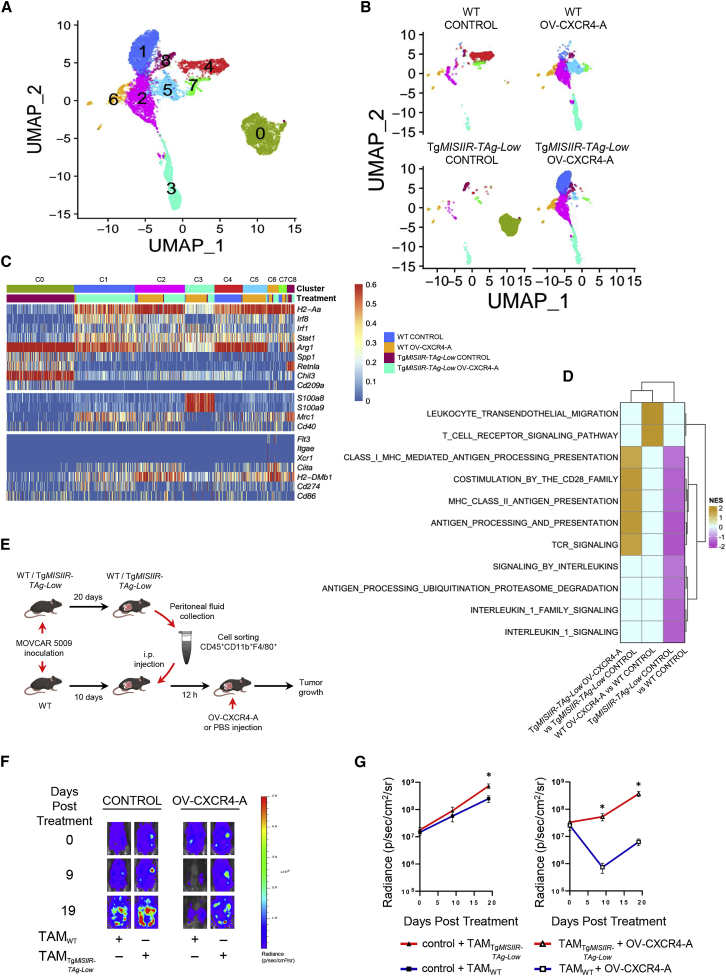
Transcriptional and functional analyses of TAMs isolated from the peritoneal TME of control and OV-CXCR4-A-treated tumors in WT and Tg*MISIIR-TAg-Low* mice (A and B) UMAP plots of re-clustered TAM subsets in the peritoneal TME all and individual control and treated MOVCAR 5009 tumors. (C) Heatmap displaying normalized expression of selected genes in each myeloid cluster in control and OV-CXCR4-A-treated tumors in syngeneic WT and Tg*MISIIR-TAg-Low* mice. (D) Heatmap of GSEA of Tg*MISIIR-TAg-Low* OV-CXCR4-A versus Tg*MISIIR-TAg-Low* control, WT OV-CXCR4-A versus WT control, and Tg*MISIIR-TAg-Low* control versus WT control in TAMs, showing normalized enrichment score (NES). (E) Graphical summary of immunosuppressive activity of TAMs (CD45^+^CD11b^+^F4/80^+^) isolated from peritoneal fluid of MOVCAR 5009 tumors in control WT and Tg*MISIIR-TAg-Low* mice (n = 10) and injected i.p. (1 × 10^6^ cells) into tumor-challenged control and OV-CXCR4-A-treated WT mice 12 h before OV-CXCR4-A treatment. Tumor-bearing mice treated with PBS served as controls. (F and G) Tumor progression was monitored by bioluminescence and presented as the mean ± SEM. ∗p < 0.05.

To better understand the heterogeneity of the TAM subsets identified through cluster analysis, we isolated and re-clustered the macrophage populations (Figures 6A, S6A, and S6B). Of the nine clusters identified, C0 and C4 were unique for untreated tumors in transgenic and WT mice, respectively (Figure 6B). Macrophages in control MOVCAR 5009-bearing WT mice, which assembled in C4, had elevated expression of M1 markers (*H2-Aa*, *Irf8*, *Irf1*, *Stat1*, and *H2-DMb1*) and low levels of some M2 markers except for *Arg1*, *Chil3*, and *Cd274* (Figure 6C). In contrast, TAMs in C0 of untreated tumors in Tg*MISIIR-TAg-Low* mice exhibited a highly immunosuppressive transcriptional profile with elevated *Arg1* and *Chil3* in parallel with other M2 markers, such as *Spp1*, *Retnla*, and *Cd209a*, along with uniformly low expression of *H2-Aa*, *Irf8*, *Irf1*, *Stat1*, and *H2-DMb1* (Figure 6C)*.* The highly immunosuppressive C0 was ablated after OV-CXCR4-A treatment in Tg*MISIIR-TAg-Low* mice. Instead, the TAM transcriptome was enriched with genes encoding M1 markers, with the exception of *Arg1* and *Mrc1*, which were concentrated in C1 and C2 (Figure 6C). Other clusters, including C3, consisted of cells with high expression of *S100A8/9* genes of low-molecular-weight intracellular calcium-binding proteins, which represent one of the hallmarks that distinguish M-MDSCs from monocytes.^41^ C5 was unique to TAMs of OV-CXCR4-A-treated WT mice, with a signature similar to that of C1. These transcriptomic differences between TAMs of control and OV-CXCR4-treated Tg*MISIIR-TAg-Low* mice were further confirmed by a gene set enrichment analysis (GSEA) performed to define the functional changes in TAMs across the treatment conditions. As depicted in Figure 6D, OV-CXCR4-A treatment of transgenic mice increased the expression of genes that are known to be involved with antigen presentation and co-stimulation of lymphoid cells, which were largely downregulated in untreated tumors of Tg*MISIIR-TAg-Low* mice.

Among other subsets analyzed, DCs represented a small fraction of myeloid cells that were concentrated mainly in C6 with expression of *Flt3*, *Itgae*, and *Xcr1* (Figure 6C). As in TAMs, the DC population in MOVCAR 5009-challenged Tg*MISIIR-TAg-Low* mice had reduced expression of *H2-Aa* (Figure S7), which was increased by OV-CXCR4-A treatment to levels comparable with those measured in the WT counterparts. We also observed enhanced expression of the inhibitory receptor Lilrb4a^42^ and Epstein-Barr virus-induced gene (*Ebi3*) implicated in regulation of Th17 and Treg cells^43^ in DCs of tumor-bearing Tg*MISIIR-TAg-Low* mice after OV-CXCR4-A treatment (Figure S7).

Based on scRNA-seq analysis, we presumed that TAMs in untreated *TgMISIIR-TAg-Low* mice have an increased capacity to suppress the antitumor immune response compared with the same cells in WT mice. To test this assumption, we examined how the differences in phenotypes and transcriptional signatures of TAMs in MOVCAR 5009-challenged control mice of the WT and the transgenic background relate to their functional activities in adoptive cell transfer (ACT) experiments to tumor-bearing WT mice. As depicted in Figure 6E, CD45^+^CD11b^+^F4/80^+^ cells were isolated from single-cell suspensions of peritoneal fluid of MOVCAR 5009-bearing WT and Tg*MISIIR-TAg-Low* mice by cell sorting (Figure S8) and injected i.p. (10^6^ cells/mouse) into tumor-challenged WT mice 12 h before OV-CXCR4-A treatment. Progression of tumor growth, quantified by bioluminescence imaging (Figure 6F), showed higher protumorigenic activity of adoptively transferred TAMs derived from Tg*MISIIR-TAg-Low* mice compared with their WT counterparts (Figure 6G). The adoptively transferred TAMs derived from Tg*MISIIR-TAg-Low* mice significantly augmented the tumor load in untreated mice (Figure 6G, left panel; day 19: p = 0.03), and the effect was even more prominent in OV-CXCR4-A-treated counterparts (Figure 6G, right panel; days 9 and 19: p = 0.03 and p = 0.01, respectively). In contrast, tumor progression after ACT of TAMs isolated from untreated WT mice was similar to that observed in animals that did not receive any adoptive transfer of TAMs (data not shown).

### Heterogeneous and functionally divergent CAFs in MOVCAR 5009-challenged WT and Tg*MISIIR-TAg-Low* mice

**Figure 7:**
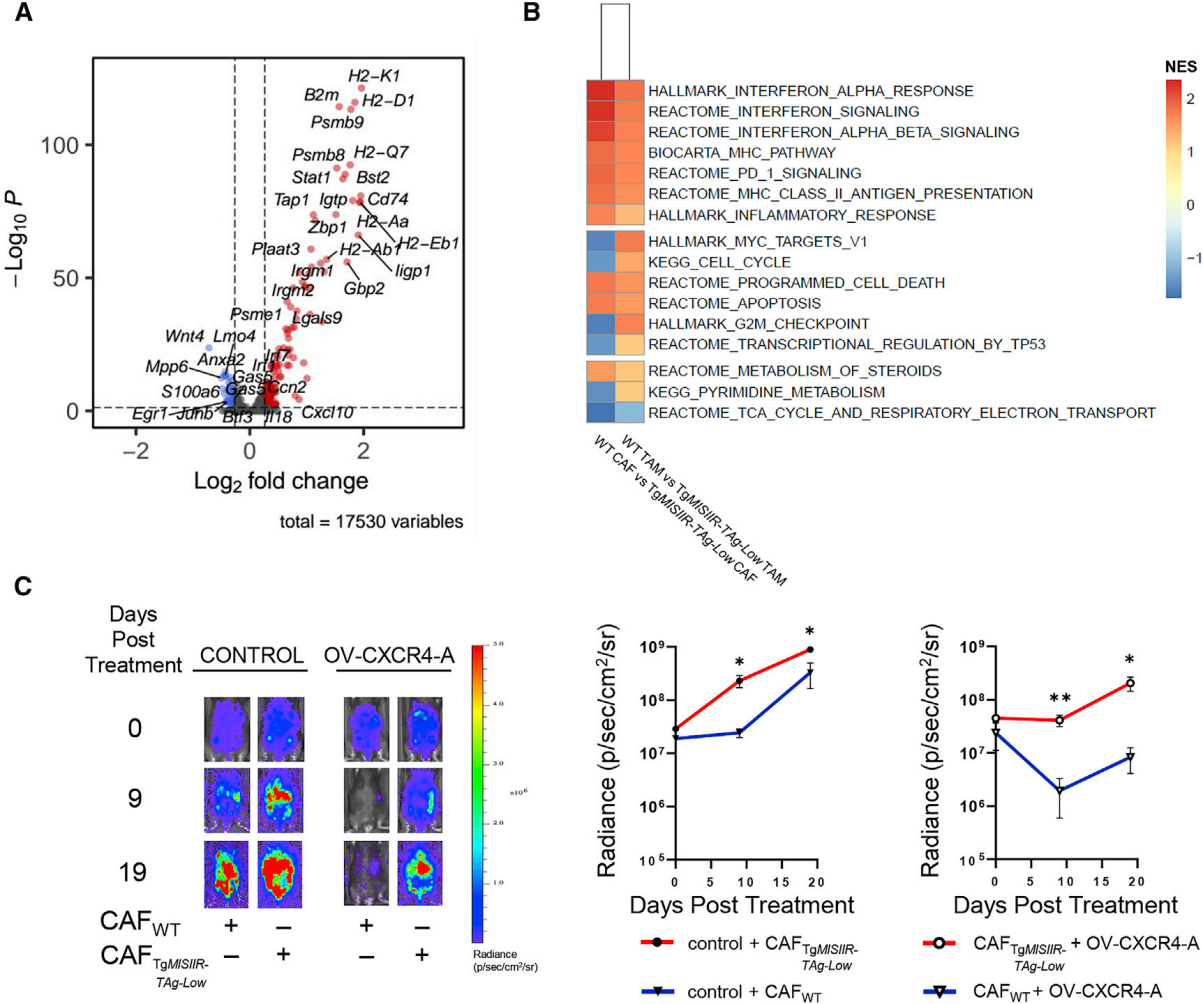
Functional and transcriptional dichotomies of CAFs in MOVCAR 5009 tumor-bearing WT and Tg*MISIIR-TAg-Low* mice (A) Volcano plot showing enrichment of differentially expressed genes in CAF clusters between control tumors of WT and Tg*MISIIR-TAg-Low* mice. Each red and blue dot denotes an individual gene with a Benjamini-Hochberg-adjusted p < 0.05 and fold change >1.2. (B) Heatmap of GSEA of WT CAFs versus Tg*MISIIR-TAg-Low* CAFs and WT TAMs versus Tg*MISIIR-TAg-Low* TAMs, showing NES. (C) Measuring the immunosuppressive activity of CAFs isolated from single-cell suspensions of peritoneal fluid 20 days after tumor challenge (n = 10 mice) of control WT or Tg*MISIIR-TAg-Low* mice. The cells (>90% CD49e^+^, <5% CD31^+^, and <3% CD45^+^) were injected i.p. (1 × 10^6^ cells) into WT mice 1 day after MOVCAR 5009 tumor challenge. Nine days later, the tumor-challenged mice were treated i.p. with OV-CXCR4-A or PBS. Tumor progression was monitored by bioluminescence (left panel), and tumor volume curves were generated, with data points representing mean ± SEM of 5 mice/group (right panel). ∗p < 0.05, ∗∗p < 0.01.

Differential gene expression analysis and GSEA of peritoneal CAFs isolated from MOVCAR 5009 tumor-challenged untreated or OV-CXCR4-A-treated WT and transgenic mice revealed dissimilarities in the transcriptional profiles of genes that are involved in immune responses and cell cycle pathways (Figures 7A and 7B). In WT mice, the CAF transcriptome was enriched in genes encoding protein and transcription factors that foster an immune-permissive TME, including enrichment for interferon (IFN)-responsive genes encoding for MHC proteins and chemoattractants, characteristic of IFN-licensed CAFs (ilCAFs).^44^ In contrast, CAFs in Tg*MISIIR-TAg-Low* mice expressed genes encoding protein and transcription factors involved in cancer progression by regulating the dynamics of cytoskeletal constituents (*S100a6* and its binding partner, Anx2),^45^^,^^46^ activating the Wnt signaling pathway involved in cancer stemness and therapy resistance (*Wnt4*),^47^ and contributing to mutant p53 gain of function (an *Erg1* transcription factor implicated in growth control and apoptosis).^48^ This profile of gene expression was associated with upregulated signaling pathways associated with the cell cycle, transcriptional regulation by *TP53*, tricarboxylic acid (TCA), and pyrimidine metabolism (Figure 7B). The dichotomies between CAF transcriptomes were also reflected in their tumorigenic activities examined in the ACT to MOVCAR 5009-challenged WT mice. As depicted in Figure S9, peritoneal CAFs were isolated from tumor-bearing WT or Tg*MISIIR-TAg-Low* mice by negative selection using the CD45^+^ column, and recovered cells consisting of over 88% of CD49e^+^ FAP^+^ CAFs were injected i.p. into WT mice 1 day after tumor inoculation, followed by OV-CXCR4-A treatment 9 days later. Progression of tumor growth, presented in Figure 7C, left panel, revealed that CAFs derived from tumor-challenged transgenic mice exhibited higher protumorigenic activities compared with their WT counterparts because a significant increase in tumor load was observed on days 9 and 19 in control (Figure 7C, right panel; p = 0.02 and p = 0.03, respectively) as well as OV-CXCR4-A-treated mice (p = 0.007 and p = 0.02, respectively).

### OV-CXCR4-A-induced expression profiling and cytotoxic activities of CD8^+^ TALs isolated from WT and Tg*MISIIR-TAg-Low* mice

**Figure 8:**
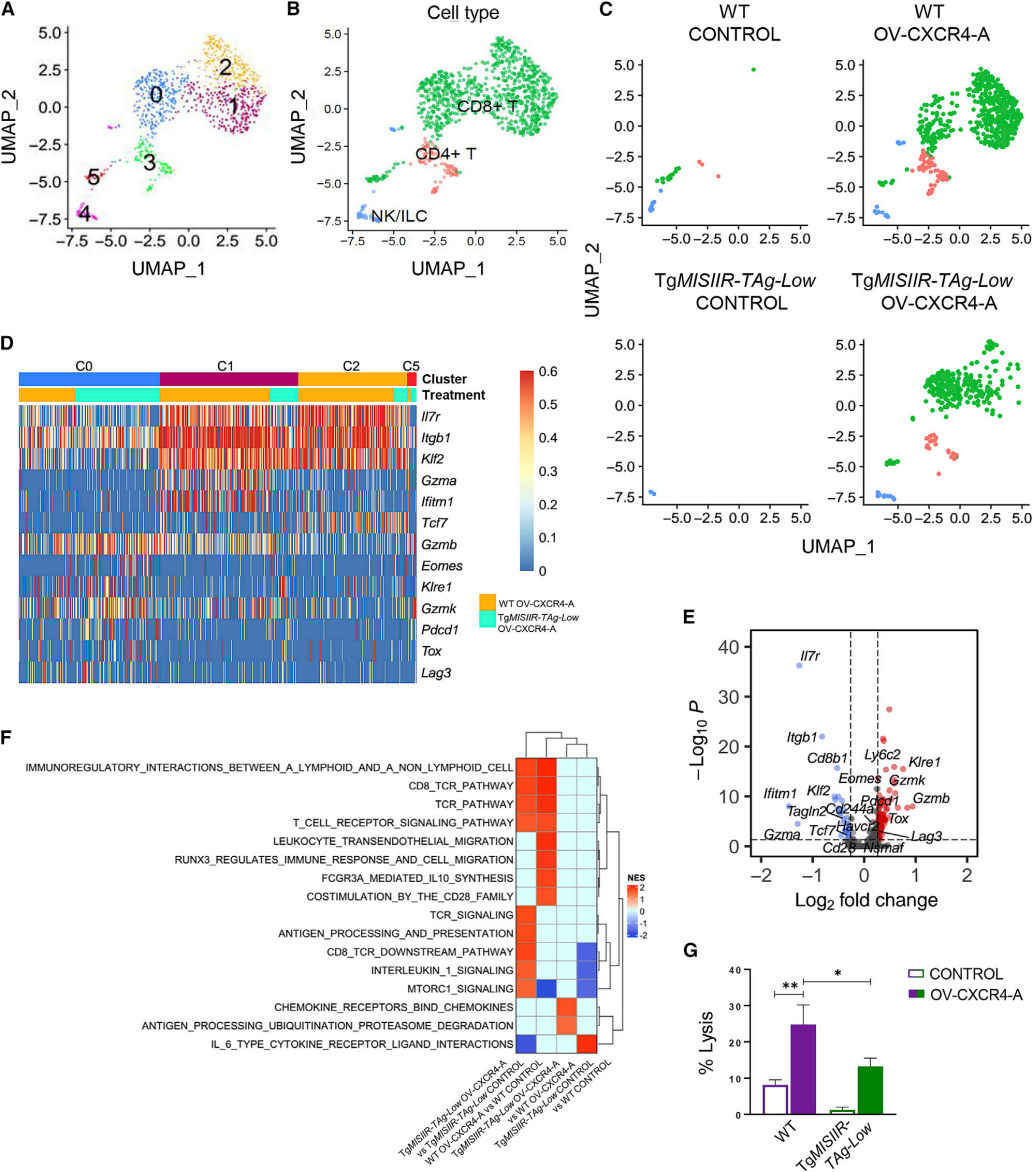
Analyses of TALs in control and OV-CXCR4-A-treated MOVCAR 5009 tumors in WT and Tg*MISIIR-TAg-Low* mice (A–C) UMAP plots of re-clustered TAL subsets in all, control, and OV-CXCR4-A-treated tumors in WT and Tg*MISIIR-TAg-Low* mice. (D) Heatmap displaying normalized expression of selected genes in each CD8^+^ T cell cluster in OV-CXCR4-A-treated tumors. (E) Volcano plot showing enrichment of differentially expressed genes in CD8^+^ T cell clusters between OV-CXCR4-A-treated tumors in Tg*MISIIR-TAg-Low* and WT mice. Each red and blue dot denotes an individual gene with a Benjamini-Hochberg-adjusted p < 0.05 and fold change > 1.2. (F) Heatmap of GSEA of Tg*MISIIR-TAg-Low* OV-CXCR4-A versus Tg*MISIIR-TAg-Low* control, WT OV-CXCR4-A versus WT control, Tg*MISIIR-TAg-Low* OV-CXCR4-A versus WT OV-CXCR4-A, and Tg*MISIIR-TAg-Low* control versus WT control in CD8^+^ TALs, showing NES. (G) CD8^+^ T cell cytotoxic activity presented as the percentage of lysis of target MOVCAR 5009 cells in control and OV-CXCR4-A-treated WT and Tg*MISIIR-TAg-Low* mice. Results are presented as mean ± SD of 5 mice/group. ∗p < 0.05, ∗∗p < 0.01.

Consistent with the flow cytometry analysis, the scRNA-seq profiling demonstrated increases in CD4^+^ and CD8^+^ TALs after OV-CXCR4-A treatment (Figures 8A–8C). In OV-CXCR4-A-treated tumors, CD8^+^ TALs were organized across four main clusters (C0, C1, C2, and C5), whereas CD4^+^ TALs were concentrated in cluster C3 (Figures 8B, S10A, and S10B). C4 consisted of NK/innate lymphoid cell (ILC) cells with elevated *Ccl3*, *Xcl1*, *Prf1*, and *Il5* gene expression (data not shown). Because of insufficient numbers of CD4^+^ and NK/ILC cells, expression profiling was performed on CD8^+^ T cells in OV-CXCR4-treated tumors and depicted in individual clusters of the heatmap (Figure 8D). CD8^+^ T cells in the peritoneal TME of WT mice were concentrated in the most prominent C1 and C2 with elevated expression of genes that are involved in regulation/maintenance of T cell homeostasis (*Klf2* and *Itgb1*), differentiation and survival (*Tcf7*), activation/maintenance of effector cells (*Ifitm1* and *Il7r*), and effector function of cytotoxic T lymphocytes (CTLs) (*Gzma*). In comparison, expression of these genes in C0 that comprised high numbers of CD8^+^ T cells from transgenic mice was lower, except for *Gzmb*, which could also indicate their exhaustion status when co-expressed with eomesodermin (EOMES) and PD1 antigens.^49^ In line with the latter possibility, C0 exhibited consistently higher expression of genes associated with tumor-induced exhaustion (*Eomes*, *Gzmk*, *Pdcd1*, *Tox*, and *Lag3*)^50^^,^^51^^,^^52^^,^^53^ as well as the *Klre1* inhibitory receptor gene.^54^ Consistently, the differential gene expression (DGE) analysis demonstrated increases in *Gzmk*, *Gzmb*, *Pdcd1*, *Tox*, and *Ly6c2* gene expression in parallel with downregulation of *Gzma*, *Il7r*, *Itgb1*, *Klf2*, and *Ifitm1* in CD8^+^ T cells of transgenic mice (Figure 8E). Similarly, the GSEA performed across different conditions revealed that OV-CXCR4-A treatment activated CD8/T cell receptor (TCR) signaling pathways, interleukin-1 and mammalian target of rapamycin (mTOR) signaling in CD8^+^ T cells derived from transgenic mice, and additional cell migration pathways in their WT counterparts (Figure 8F).

To examine whether changes in the transcriptomic signatures of CD8^+^ TALs correlated with their functional activities, we performed cytotoxic assays against MOVCAR 5009 cells. CD8^+^ T cells isolated from single-cell suspensions of peritoneal fluid of control and OV-CXCR4-A-treated WT and Tg*MISIIR-TAg-Low* mice were incubated with CellTracker Blue CMF_2_HC-labeled MOVCAR 5009 target cells at a 1:1 ratio for 17 h, and numbers of live cells were determined by flow cytometry. As depicted in Figure 8G, CD8^+^ T cells isolated from OV-CXCR4-A-treated transgenic mice exhibited reduced cytotoxic activity compared with their counterparts from tumor-bearing WT mice (p = 0.03), which is consistent with increased expression of genes associated with tumor-induced exhaustion and a reduced level of TCR_Tag-I_ tetramer binding. Furthermore, ∼4% of CD8^+^ TALs in the peritoneal TME of OV-CXCR4-A-treated transgenic mice co-expressed CD38 and CD101 antigens (Figures S11A and S11B; p = 0.012) associated with a fixed state of dysfunction,^55^^,^^56^ suggesting that the responses were less sustainable compared with those in WT mice.

## Discussion

We demonstrated that targeting the interaction between immunosuppressive CAFs and TAMs in the tolerogenic TME by OV-CXCR4-A induces tumor/self-specific CD8^+^ T cell responses that participate in tumor control. The transcriptomic and functional analyses revealed that, while the majority of CD8^+^ TALs in Tg*MISIIR-TAg-Low* mice preserved some of the genetic signatures of effector cells, they expressed genes characteristic of dysfunctional tumor-specific CD8^+^ T cells and were less effective in controlling tumor growth compared with their counterparts in WT mice. This suggests that the unresponsive tumor-specific CD8^+^ T cells in MOVCAR 5009-challenged Tg*MISIIR-TAg-Low* mice were reprogrammed by OV-CXCR4-A-induced epitope spreading and exposure to TAg-specific activation, although they remained in a hypofunctional effector stage characterized by upregulation of *Tox* along with inhibitory receptors, including *Lag3* and *Pdcd1*.^51^ These findings are consistent with previous reports demonstrating that immune tolerance to self can be induced by vaccination with cognate antigen(s) that alters the previously fixed transcriptional signature and increases antigen-sensing machinery.^3^^,^^4^^,^^57^^,^^58^ From a mechanistic standpoint, however, our studies emphasize the importance of the CAF-TAM cross-talk in regulating tumor growth as well as induction of antitumor immunity in the setting of immune tolerance to tumor/self-antigens. Although the contribution of peritoneal M2 TAMs to tumor growth in transgenic mice is consistent with the results of a meta-analysis performed on 794 OC patients showing a positive correlation between M2-TAMs in tumors and low overall survival,^25^ the tolerance-prone TME was not solely influenced by the M2 macrophages because targeting CAFs by OV-CXCR4-A was critical in reversing their immunosuppressive landscape. These findings, together with the accelerating tumor growth after adoptive transfer of immunosuppressive CAFs to MOVCAR-bearing WT mice, stress their pivotal role in maintaining a pro-tumoral niche and regulating complex tumor-stroma interaction.^27^^,^^59^ Furthermore, it also appears that the transcriptional and functional dichotomies of CAFs in MOVCAR 5009-bearing WT and transgenic mice were less dependent on FAP and PDPN antigen expression because both markers were detected on pro-inflammatory and protumorigenic CAFs, emphasizing a challenge in their targeting strategies during *in vivo* tumor evolution.^60^

The highly immunosuppressive nature of CAFs in Tg*MISIIR-TAg-Low* mice raises the possibility that they could influence the generation and maintenance of M2 TAMs, which dominated the landscape of peritoneal tumors in transgenic mice and could suppress CD8^+^ T cell proliferation and activation in a cell-cell contact-dependent manner (reviewed in Pan et al.^61^). Thus, it seems plausible that their depletion in MOVCAR 5009-bearing transgenic mice could be responsible for the therapeutic effect of OV-CXCR4-A treatment, resulting in proinflammatory repolarization of M2 TAMs and activation of TAg-specific CD8^+^ T cells. Although the mechanisms responsible for the nearly complete depletion of CAFs by OV-CXCR4-A are not fully understood, we hypothesize that they could be related to apoptosis of CXCR4-expressing CAFs after binding to the CXCR4 antagonist released from OV-CXCR4-A-infected tumor cells, as reported previously.^11^ A similar effect on repolarization of M2 ascites macrophages by targeting CAFs with oncolytic group B adenovirus enadenotucirev expressing a stroma-targeted bispecific T cell engager has been reported.^62^ These observations were also supported by the lack of an immunosuppressive effect of CAFs isolated from tumor-bearing WT mice, which were characterized by expression of genes encoding protein and transcription factors that foster an immune-permissive TME. Therefore, we anticipate that additional epigenomic profiling studies will provide some insight into the epigenetic processes in CAFs from tumor-bearing WT and Tg*MISIIR-TAg-Low* mice and help to identify potential avenues to reprogram CAFs to a state that is not supportive of cancer invasion or immune suppression and instead supports local immune activation. Such a comparison would be particularly useful for rational designs of combination therapy regimens. Especially the limited benefit from immune checkpoint blockade when used in monotherapy or combination therapy for OC^63^^,^^64^ stresses the need for new therapeutic approaches combined with an improved understanding of T cell biology in the context of the tolerogenic TME.

Our finding that a MOVCAR 5009 tumor implanted in WT mice does not recapitulate the tolerogenic model of tumor-bearing Tg*MISIIR-TAg-Low* counterparts is consistent with previous studies that identified poor concordance between the syngeneic and autochthonous model of breast cancer.^65^ The availability of WT and tolerogenic OC syngeneic murine models allowed mechanistic exploration of some questions underlying the unique transcriptional programs of CAFs between these two groups of mice as well as their interaction with TAMs. Because orthotopic implantation of MOVCAR 5009 cells resulted in dissemination of tumor cells through the abdominal cavity, accompanied by production of malignant ascites in both groups of mice, the increased tumorigenicity in tumor/self-antigen expressing transgenic mice could be explained, at least in part, by an impaired ability to generate antitumor immune responses because of tolerance. However, it is also possible that the sites of tumor implantation generate a niche that is more or less conducive to the tumor initiation process. For example, although the oncogenic TAg is not directly associated with development of human cancer, its expression at the tumor site could result in functional inactivation of the critical tumor suppressor p53 and retinoblastoma (Rb) pathways,^22^ leading to production of a variety of tumor-derived factors that facilitate accumulation of immunosuppressing CAFs without forming a tumor. In humans, mutation of *TP53* is by far the most common genetic alteration observed in OC, and direct mutation or loss of *Rb* or its downstream signaling mediators is also common in human OCs.^66^ Via binding and inhibition of PP2A, TAg also results in activation of phosphatidylinositol 3-kinase (PI3K)/AKT (serine/threonine-protein kinase) and mitogen-activated protein kinase (MAPK) signaling,^67^ which are frequently activated in human OC.^68^ Thus, the availability of MOVCAR 5009 cells exhibiting genetic alterations pertinent to human OC together with the non-tumor prone Tg*MISIIR-TAg-Low* mice expressing the TAg antigen at the tumor site allow studying the *in vivo* effects of oncogenic changes in the tumorigenic niche on the interactions between cancer cells and the TME in an immunocompetent host.

Our observations underscore the potential utility of Tg*MISIIR-TAg-Low* mice for preclinical evaluation of therapeutic agents. For example, the presence and precise functions of TAMs and CAFs are complex and incompletely understood, in part because of a lack of comprehensive studies describing the interplay between these cells as well as variable results with efforts to target CAFs or TAMs individually. Instead, targeting the CAF-TAM interaction by CXCR4-A-armed oncolytic virotherapy may hold significant potential to improve the outcome of cancer treatment when rationally combined with other treatment modalities. In this context, tumor-selective oncolytic viruses are likely to provide a means to improve multiple therapies by targeted destruction of the TME to enhance the infiltration and effector function of other components of the immune system. Identifying preclinical mouse models, such as that the one presented here, provides opportunities for mechanistic interrogation of the OC TME following cancer immunotherapy. Therefore, elucidating the molecular mechanisms that establish and maintain tumor antigen-driven, dysfunctional differentiation of tumor/self- and neoantigen-specific T cells has the potential to lead to new effective immunotherapeutic interventions in patients with poor responses to contemporary treatments.

## Materials and methods

### Mice and cell lines

Female WT C57BL/6 mice (6–8 weeks old) were obtained from Charles River Laboratories (Wilmington, MA, USA). The non-tumor-prone C57BL/6 Tg*MISIIR-TAg-Low* transgenic mice expressing the TAg protein in epithelial cells lining the fallopian tubes under transcriptional control of the Müllerian inhibiting substance type II receptor gene promoter^22^ were obtained from Dr. Denise Connolly (Fox Chase Cancer Center, Philadelphia, PA, USA) and bred in the Laboratory Animal Resources at Roswell Park Comprehensive Cancer Center (RPCCC) (Buffalo, NY, USA). The animal studies were performed following guidelines established by the Institutional Animal Care and Use Committee (IACUC) under an approved protocol. The TAg-expressing MOVCAR 5009 cell line, transduced with a retroviral construct encoding the firefly luciferase gene (pWZL-Luc) for *in vivo* imagining,^22^ was provided by Dr. Denise Connolly (Fox Chase Cancer Center, Philadelphia, PA, USA). This cell line was established from ascites of spontaneous ovarian tumors developed in Tg*MISIIR-TAg* mice and selected based on tumorigenicity in syngeneic Tg*MISIIR-TAg-Low* and WT C57BL/6 mice.^24^ MOVCAR 5009 cells were cultured in DMEM (Corning) supplemented with 10% fetal bovine serum (FBS; Corning) and 5 μg/mL gentamicin sulfate (Corning) and maintained at 37°C and 5% CO_2_. The MOVCAR 5009 cell line was authenticated by short tandem repeat (STR) profiling at the ATCC (Gaithersburg, MD, USA).

### Oncolytic virotherapy

The generation and characterization of oncolytic vaccinia viruses expressing EGFP (OV-EGFP) and the CXCR4 antagonist in the context of the Fc portion of murine immunoglobulin G2a (IgG2a; OV-CXCR4-A) have been described previously.^9^

### *In vivo* studies

Mice were injected i.p. with 5 × 10^6^ MOVCAR 5009 cells. Ten days after the tumor challenge, mice were treated i.p. with 5 × 10^7^ PFUs of OV-EGFP or OV-CXCR4-A. Control mice were treated with PBS. Tumor growth was monitored by bioluminescence imaging, and quantification of bioluminescence signals was performed using the in vivo imaging system (IVIS) Spectrum In Vivo Imaging System (PerkinElmer, Waltham, MA, USA). Average radiance (photons/s/cm^2^/sr) in the standard region of interest (ROI) was determined using the Living Image 4.7.3 software for IVIS Spectrum.

### Flow cytometry

Flow cytometry analysis was performed on single-cell suspensions obtained from peritoneal fluids of MOVCAR 5009-bearing mice 10 days after treatment. Red blood cells were lysed using ammonium-chloride-potassium (ACK) lysis buffer (Quality Biological, Gaithersburg, MD, USA), and cells were incubated with anti-mouse CD16/CD32 Fc blocker (BD Biosciences, San Jose, CA, USA) for 20 min at 4°C before staining with the selected cell surface markers. For intracellular assays, the Transcription Factor Buffer Set (BD Biosciences, San Jose, CA, USA) was used according to the manufacturer’s recommendation. All fluorochrome-conjugated antibodies were purchased from BD Biosciences (San Jose, CA, USA), BioLegend (San Diego, CA, USA), Sigma-Aldrich (St. Louis, MO, USA), or Thermo Fisher Scientific (Waltham, MA, USA), as detailed in Table S1. The TAg-D^b^/SAINNYAQKL tetramer conjugated to PE was obtained from MBL International (Woburn, MA, USA), and the B8R-K^b^/TSYKFESV tetramer conjugated to PE was obtained from Baylor College of Medicine (Houston, TX, USA). All samples were analyzed on the LSR Fortessa flow cytometer (BD Biosciences), and data analysis was performed using WinList 3D 9.0.1 (Verity Software House, Topsham, ME, USA). In the analysis, doublets were excluded using forward scatter width (FSC-W) versus side scatter area (SSC-A), followed by exclusion of dead cells stained with LIVE/DEAD Fixable Aqua Dead Cell Stain (Invitrogen, Waltham, MA, USA).

### T cell depletion

To achieve sufficient depletion in mice, beginning 1 day before MOVCAR 5009 tumor inoculation, mice were injected i.p. with 100 μg/injection of anti-CD4 or anti-CD8α in 300 μL PBS, followed by the same dosing on days 2, 6, 10, and 14. Control mice received an equal dose of anti-keyhole limpet hemocyanin (KLH) as an isotype control. The anti-CD4 (clone YTS 191), anti-CD8 (clone YTS 169.4), and isotype control (clone LTF-2) antibodies were obtained from Bio X Cell (Lebanon, NH, USA). Ten days after the tumor challenge, mice were treated i.p. with 5 × 10^7^ PFUs of OV-EGFP or OV-CXCR4-A. Flow cytometry staining of peripheral blood CD8 and CD4 lymphocytes with noncompetitive anti-CD8 and anti-CD4 antibodies (Abs) was performed 1 day after the last Ab treatment to confirm reductions of the respective T cell subsets (>92%). The fluorochrome-conjugated noncompetitive anti-CD4-APC (allophycocyanin) (clone RM4-4) and anti-CD8α-fluorescein isothiocyanate (FITC) (clone CT-CD8) mAbs were purchased from BioLegend (San Diego, CA, USA) and Thermo Fisher Scientific (Waltham, MA, USA), respectively, as detailed in Table S1. Tumor growth was monitored by bioluminescence imaging.

### ACT

Donor mice, WT or Tg*MISIIR-TAg-Low*, were injected i.p. with 5 × 10^6^ MOVCAR 5009 cells, and peritoneal fluid was collected 20 days after tumor inoculation for separation of TAMs or CAFs for ACT to the recipient tumor-challenged WT mice. For the adoptive transfer of TAMs, CD45^+^CD11b^+^F4/80^+^ cells were isolated from single-cell suspensions obtained from peritoneal fluid of tumor-bearing mice after sorting using the BD FACS Aria Cell Sorter under sterile conditions. Cells that were more than 95% CD11b^+^F480^+^ were injected i.p. (1 × 10^6^) into MOVCAR 5009-challenged WT mice 10 days after tumor inoculation. Twelve hours after TAM transfer, mice were treated i.p. with OV-CXCR4-A (5 × 10^7^ PFUs) or PBS. Tumor growth was monitored by bioluminescence imaging. For the adoptive transfer of CAF, CD45^−^ cells were prepared from single-cell suspensions obtained from peritoneal fluid of tumor-bearing mice by negative selection using CD45 MicroBeads (Miltenyi Biotec, Baltimore, MD, USA) following the manufacturer’s instructions. The CD49e-enriched CAF populations (>90% CD49e^+^, <5% CD31^+^, and <3% CD45^+^) were resuspended in PBS and injected i.p. into MOVCAR 5009-challenged WT mice (1 × 10^6^ cells) 1 day after tumor inoculation. Nine days after CAF transfer, mice were treated i.p. with 5 × 10^7^ PFUs of OV-CXCR4-A or PBS. Tumor growth was monitored by bioluminescence imaging.

### Cytotoxicity assay

Effector CD8^+^ T cells were obtained from single-cell suspensions of peritoneal fluid collected from MOVCAR 5009-bearing mice 10 days after OV-CXCR4-A treatment using the CD8^+^ T cell Isolation Kit (Miltenyi Biotec, Baltimore, MD, USA). The isolated CD8^+^ T cells were mixed with MOVCAR 5009 target cells labeled with CellTracker Blue CMF_2_HC (Thermo Fisher Scientific, Waltham, MA) at a 1:1 ratio in 500 μL of DMEM supplemented with 10% FBS and incubated in triplicates at 37°C for 17 h. Cultures containing CellTracker Blue CMF_2_HC-labeled MOVCAR 5009 cells only were included as controls. After the incubation time, cultures were collected and analyzed on an LSR Fortessa flow cytometer (BD Biosciences, San Jose, CA, USA). Cell killing was calculated using the following formula: % lysis = 100% − ([% labeled cells in experimental group / % labeled target cells] × 100%).

### scRNA-seq

#### Sample preparation

Single-cell suspensions were prepared from peritoneal fluid of MOVCAR 5009-bearing WT and Tg*MISIIR-TAg-Low* mice 10 days after OV-CXCR4-A treatment. Untreated tumor-bearing mice served as controls. Red blood cells were lysed using ACK lysis buffer and washed in 0.04% BSA in PBS. Cells were stained with Cell Multiplexing Oligos from the 3′ CellPlex Kit Set (10X Genomics, Pleasanton, CA, USA) for 5 min at room temperature and washed three times with 1% BSA in PBS. Single-cell gene expression libraries were generated using the 10X Genomics Chromium Next GEM Single Cell 3′ Kit (v.3.1). Briefly, cells tagged with CellPlex oligos were first assessed with trypan blue using a Countess FL automated cell counter (Thermo Fisher Scientific, Waltham, MA, USA), to determine the concentration, viability, and absence of clumps and debris that could interfere with single-cell capture. Two samples with different CellPlex tags were then pooled together in equal amounts for multiplexing before loading into the Chromium Controller (10X Genomics, Pleasanton, CA, USA). Reverse transcription was performed, and the resulting cDNA was amplified. Amplified cDNA was separated into full-length transcript and CellPlex Barcode fractions using SPRISelect beads (Beckman Coulter, Brea, CA, USA). The full-length amplified cDNA was used to generate transcriptome libraries by enzymatic fragmentation, end repair, a-tailing, adapter ligation, and PCR to add Illumina-compatible sequencing adapters. CellPlex barcode-derived cDNA was PCR amplified to incorporate Illumina adapter sequences and unique sample indexes. The resulting libraries were evaluated on D1000 screen tape using a TapeStation 4200 (Agilent Technologies, Santa Clara, CA, USA) and quantitated using the Kapa Biosystems qPCR Quantitation Kit for Illumina. They were then pooled, denatured, and diluted to 300 pM (picomolar) with 1% PhiX control library added. The resulting pool was then loaded into the NovaSeq reagent cartridge and sequenced on a NovaSeq6000 following the manufacturer’s recommended protocol (Illumina, San Diego, CA, USA). The raw sequencing data from the 10X Genomics libraries were processed using Cellranger v.6.0.0 software with multi-function to de-multiplex and generate count files for both transcripts as CellPlex barcodes. CellPlex barcodes were used to identify different samples in the pool, and data were separated for individual samples with Cellranger multi-function.

#### scRNA-seq analyses

For the Chromium 10X Genomics libraries, the raw sequencing data, mapping results (binary alignment and map [BAM] files), and quantification matrices were generated using Cellranger v.6.0.0^69^ software with the mouse mm10 genome and GENCODE annotation database. Then the filtered gene-barcode matrices, which contain barcodes with the unique molecular identifier (UMI) counts that passed the cell detection algorithm, were used for further analysis. All downstream analyses were performed using the Seurat^70^ single-cell data analysis R package. First, cells with very low or high RNA feature content (<500 or >7500 genes detected) or higher mitochondrial RNA content (>15%) were filtered out from the analysis to remove empty cells and doublets. Then, the normalized and scaled UMI counts were calculated using the SCTransform method. Subsequently, dimension reductions, including principal-component analysis (PCA), UMAP, and t-distributed stochastic neighbor embedding (tSNE), were carried out using the highly variable genes. Cell clusters were identified using the shared nearest neighbor (SNN)-based clustering on the first 12 principal components. The cell clusters were annotated by SingleR packages^71^ using the ImmGen reference database of the celldex R package.^71^ The M1 and M2 macrophage scores were calculated using the AddModuleScore function from the Seurat package with M1 and M2 macrophage signature genes. Differentially expressed genes between clusters and samples were identified using the FindMarkers function with the Wilcoxon rank-sum test from Seurat. Pathway analysis was carried out using the fgsea R package^72^ with the gene list ranked by average log2 fold change. The volcano plots were generated with the EnhancedVolcano R package,^73^ and the heatmaps were generated using the heatmap R package.^74^

#### Pathway analyses

GSEA of select differential expression profiles identified between groups or clusters was done using enrichR and clusterProfiler in R. Single-cell functional enrichment analysis was done using AUCell,^75^ which applies an area under the curve method to query cell-to-cell pathway activity that is robust to noise typical of scRNA-seq datasets. Six pathway databases (Hallmark Pathways, Gene Ontology [GO] biological processes, BioCarta, Kyoto Encyclopedia of Genes and Genomes (KEGG), Reactome, and the Pathway Interaction Database [PID]) were compiled from the Molecular Signatures Database (MSigDB)^76^ and used as reference sets for functional enrichments. For GSEA, only gene sets with p < 0.05 and false discovery rate (FDR) q < 0.25 were considered significantly enriched. To visualize select functional enrichments, we generated heatmaps of normalized enrichment scores of relevant biological pathways.

### Statistical analysis

All statistical analyses were performed using GraphPad Prism 6 (GraphPad, San Diego, CA, USA). Unless otherwise noted, data are presented as mean ± S.D., combined with an unpaired, two-tailed Student’s t test. Kaplan-Meier survival plots were prepared, and median survival times were determined for tumor-challenged groups of mice. Statistical differences in survival across groups were assessed using the log rank Mantel-Cox method. The threshold for statistical significance was set to p < 0.05.

## Data availability

The raw data and quantification matrices of scRNA-seq have been deposited in the database of Gene Expression Omnibus (GEO) under accession number GSE199880. The data supporting the findings reported in this study are available within the manuscript and supplemental information.

## Supplemental information

Document S1. Figures S1–S11 and Table S1

Document S2. Article plus supplemental information
