# Supplementary material for: Reprogramming the tumor microenvironment leverages CD8+ T cell responses to a shared tumor/self antigen in ovarian cancer
Source: Mol Ther Oncolytics. 2023 Feb 9;28:230–48. doi: 10.1016/j.omto.2023.02.002 (PMC9982455; doi:10.1016/j.omto.2023.02.002)
Supplement: Document S1. Figures S1–S11 and Table S1 [file mmc1.pdf]

OMTO, Volume *28*

Supplemental information

Reprogramming the tumor microenvironment leverages CD8^{+} T cell responses to a shared tumor/self antigen in ovarian cancer

Anna Mistarz, Marta Winkler, Sebastiano Battaglia, Song Liu, Alan Hutson, Hanna Rokita, Andrea Gambotto, Kunle O. Odunsi, Prashant K. Singh, A.J. Robert McGray, Jianmin Wang, and Danuta Kozbor

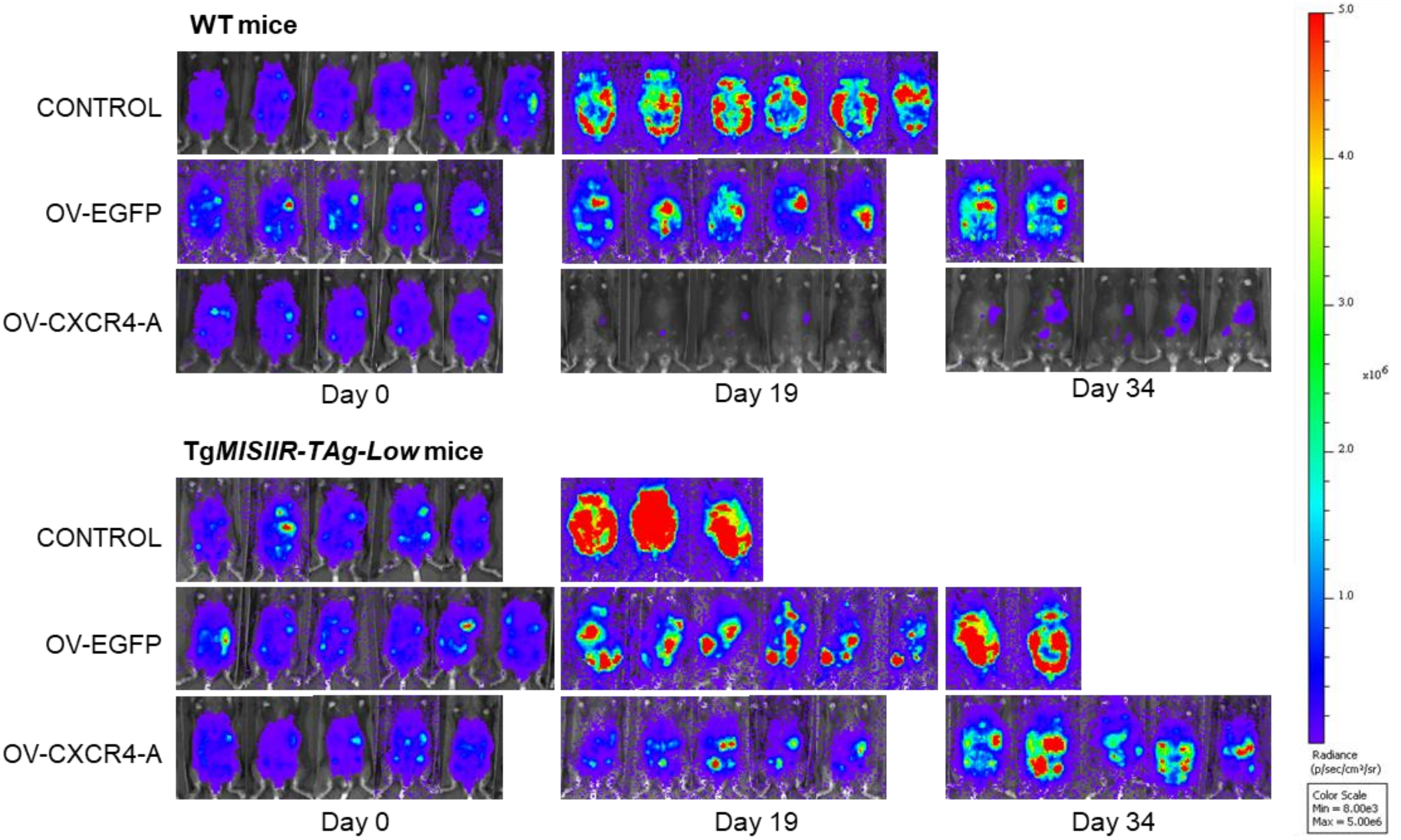

Figure S1. The effect of OV-EGFP and OV-CXCR4-A treatment on the progression of MOVCAR 5009 tumor growth in WT and Tg*MISIIR-TAg-Low* mice. Tumor progression was monitored by bioluminescence imaging on days 0, 19, and 34 (*n* = 5 - 6 mice per group).

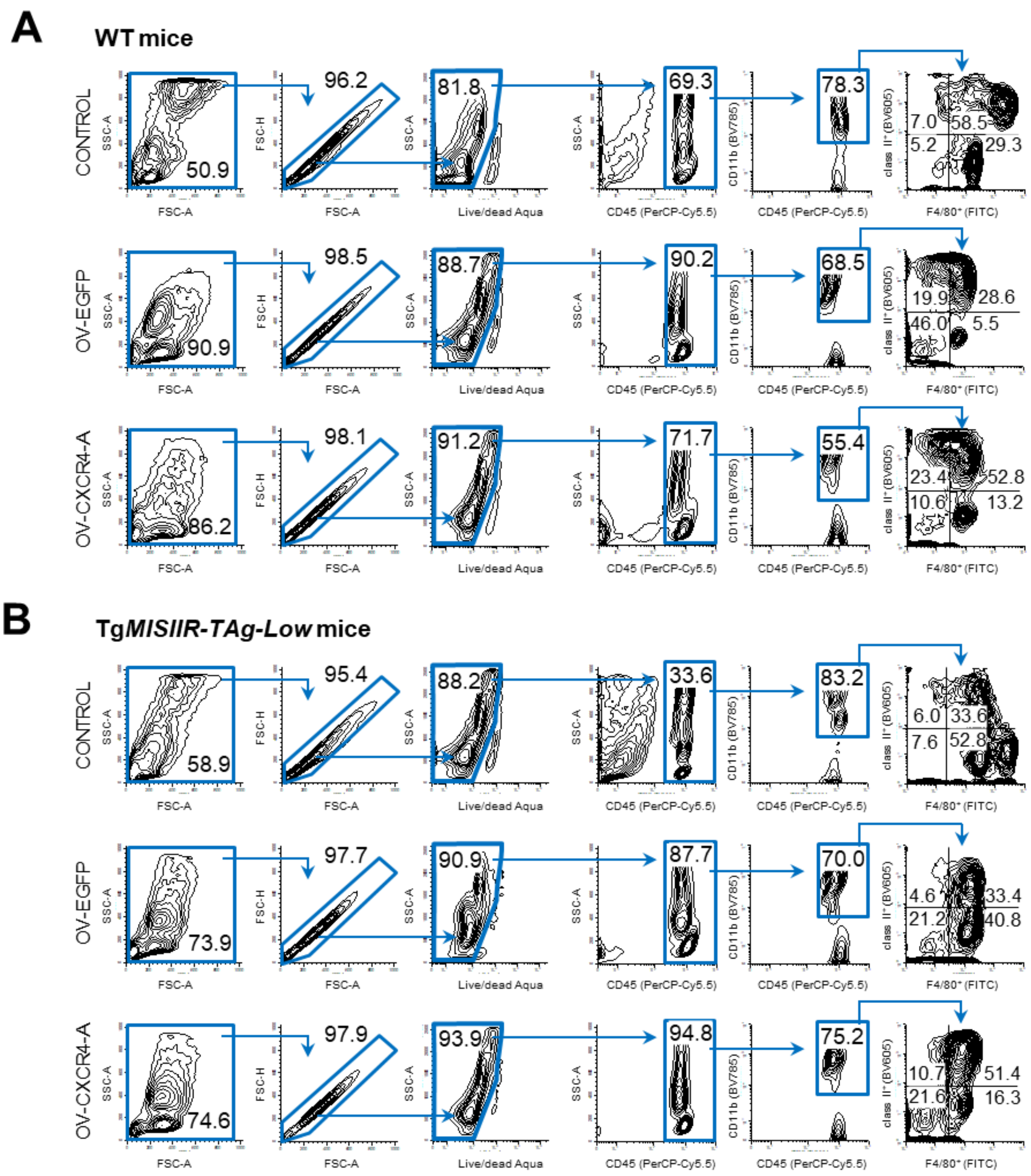

Figure S2. Gating strategy used to subset TAM populations in peritoneal TME of MOVCAR 5009-bearing mice. Representative flow cytometric plots showing differences in MHC class II expression among F4/80⁺ TAMs in control, OV-EGFP- and OV-CXCR4-A-treated tumors in WT (**A**) and transgenic (**B**) mice.

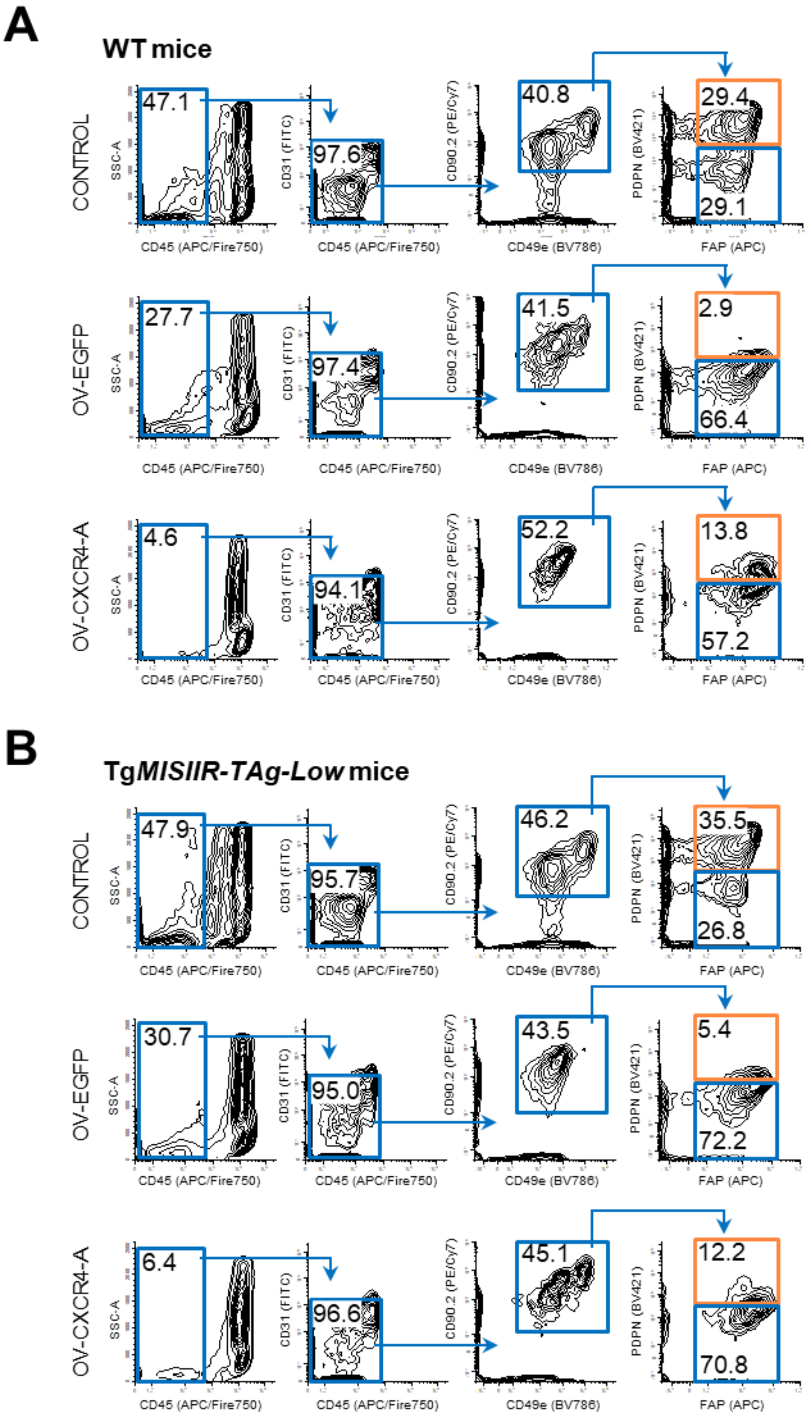

Figure S3. Staining of CAFs in peritoneal TME of WT and Tg*MISIIR-TAg-Low* mice. Representative staining of CAFs in MOVCAR 5009-challenged control, OV-EGFP- and OV-CXCR4-treated WT (**A**) and Tg*MISIIR-TAg-Low* (**B**) mice analyzed 10 days after the virotherapy treatment. Single-cell suspensions prepared from peritoneal fluids of tumor-bearing mice were stained with mAbs specific for CD45 and CD31, and cells negative for both antigens were analyzed for expression of CD49e and CD90.2. The CD49e^{+}CD90.2^{+} cells were gated and analyzed for expression FAP and PDPN.

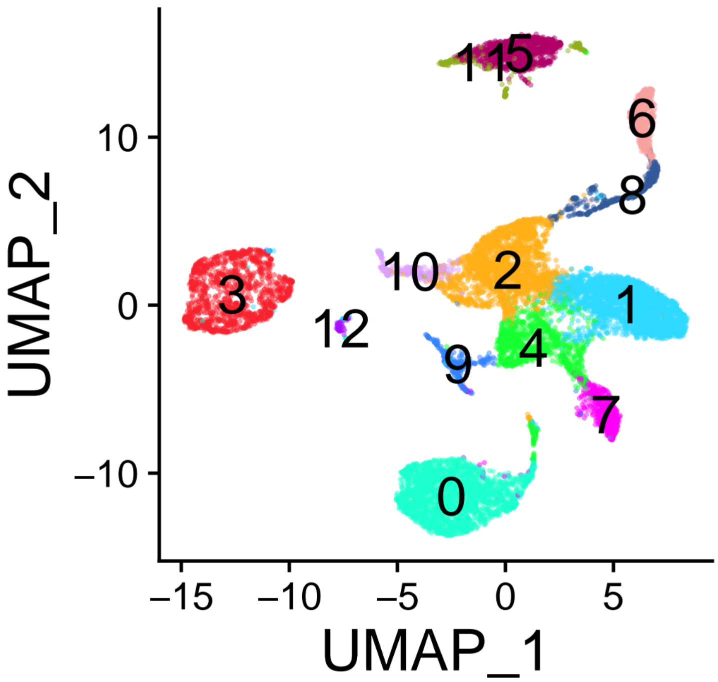

Figure S4. Clustering all single cells isolated from the peritoneal fluids of MOVCAR 5009 ovarian tumor-bearing untreated and OV-CXCR4-A-treated WT and Tg*MISIIR-TAg-Low* mice.

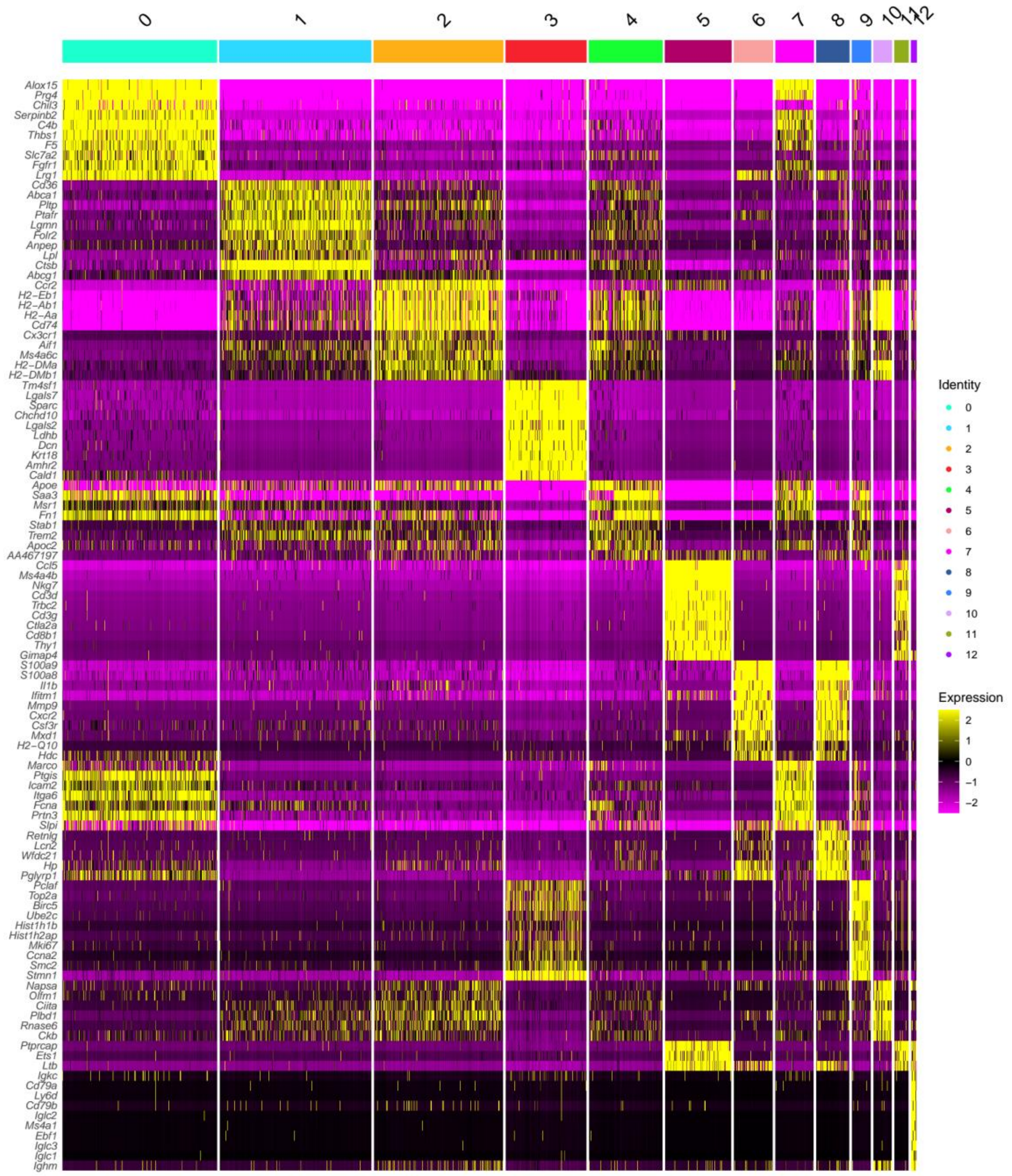

Figure S5. Heatmap of genes of lymphoid and non-lymphoid cells isolated from the peritoneal fluids of MOVCAR 5009 ovarian tumor-bearing untreated and OV-CXCR4-A-treated WT and Tg*MISIIR-TAg-Low* mice. Thirteen clusters (0 to 12) were identified and the cell types for each cluster are as follows: C0, M2 TAMs; C1, M1 TAMs; C2, monocytes/M1 TAMs; C3, tumor cells/endothelial cells/fibroblasts; C4, M0 TAMs; C5, T cells/NKT; C6, M1 TAMs/neutrophils; C7, M0 TAMs; C8, neutrophils, C9, M0 TAMs; C10, DC; C11, T cells; C12, B cells.

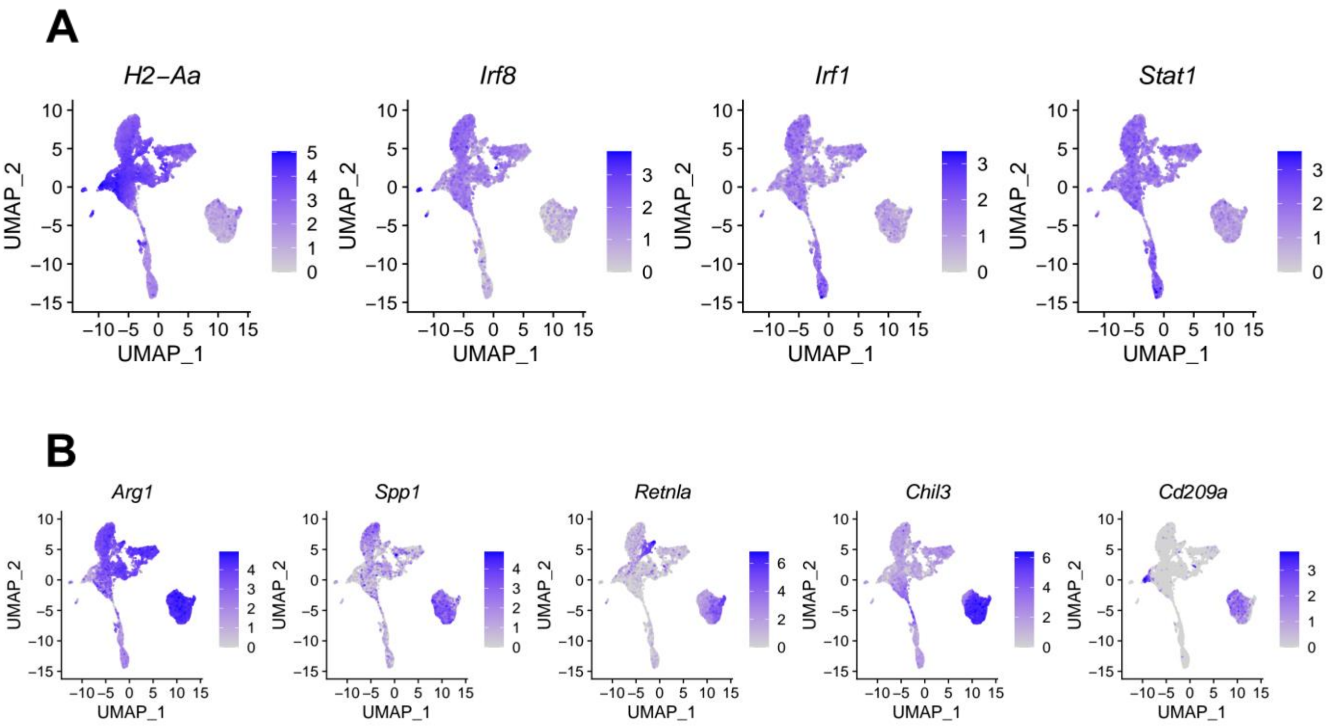

Figure S6. Transcriptional analyses of TAMs isolated from the peritoneal TME of control and OV-CXCR4-A-treated tumors in WT and Tg*MISIIR-TAg-Low* mice. UMAP plots of re-clustered TAM subsets in MOVCAR 5009 tumors were analyzed for expression of *H2-Aa, Irf8, Irf1,* and *Stat1* (**A**) and *Ag1, Spp1, Retnla, Chil3,* and *Cd209a* (**B**).

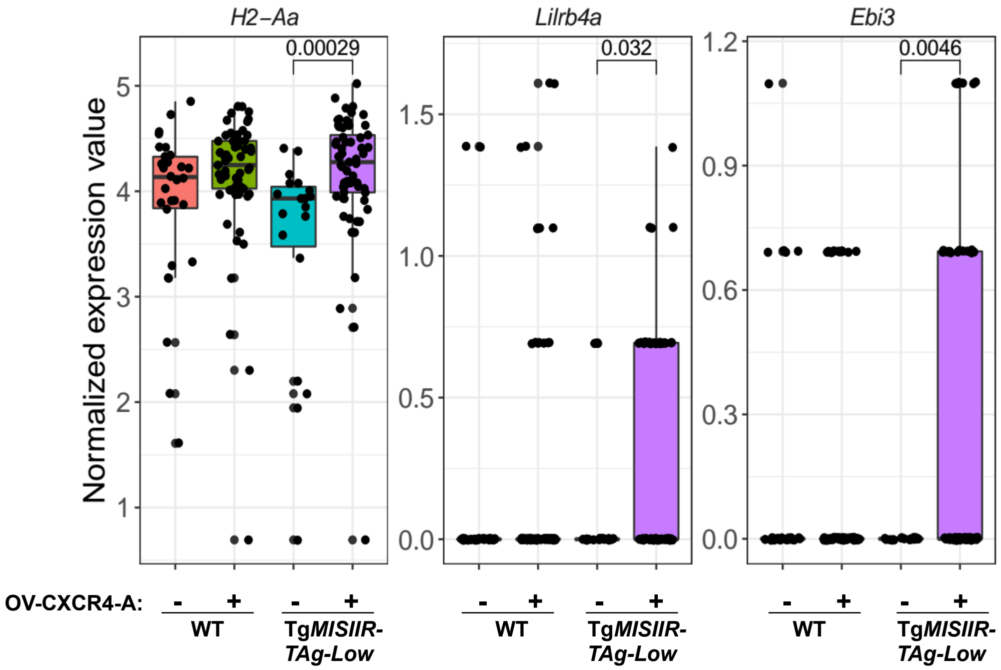

Figure S7. Analysis of DCs in the peritoneal TME of control and OV-CXCR4-A-treated WT and Tg*MISIIR-TAg-Low* mice. Bar plots displaying a normalized expression of selected genes in tumor-infiltrating DCs in control and OV-CXCR4-A-treated WT and Tg*MISIIR-TAg-Low* mice.

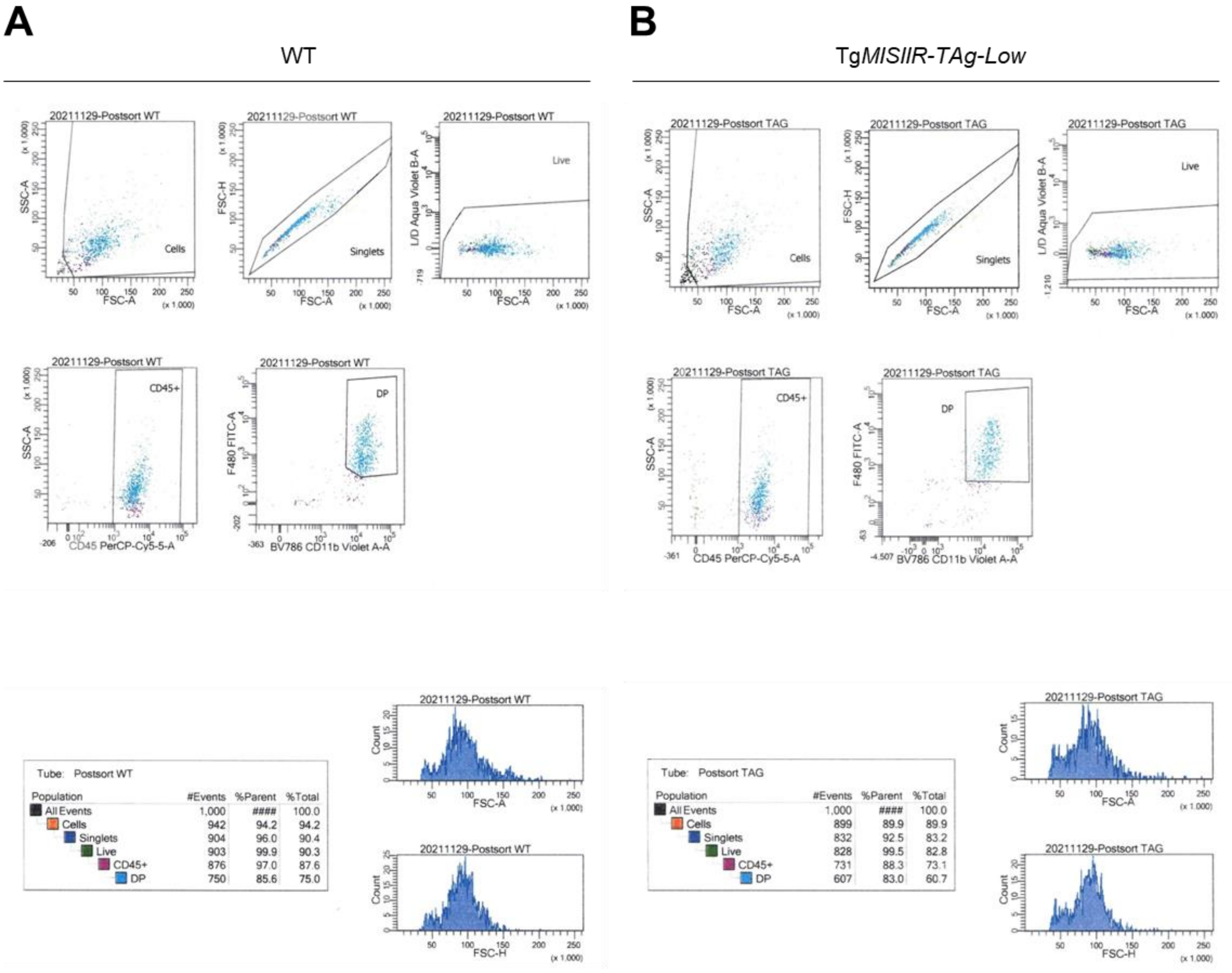

Figure S8. Flow cytometry analyses of TAMs used for adoptive cell transfer to MOVCAR 5009-challenged WT mice. Cell sorting data of TAMs ($CD45^+CD11b^+F4/80^+$) isolated from peritoneal fluids of MOVCAR 5009 tumors in control WT (**A**) and Tg*MISIIR-TAg-Low* (**B**) mice.

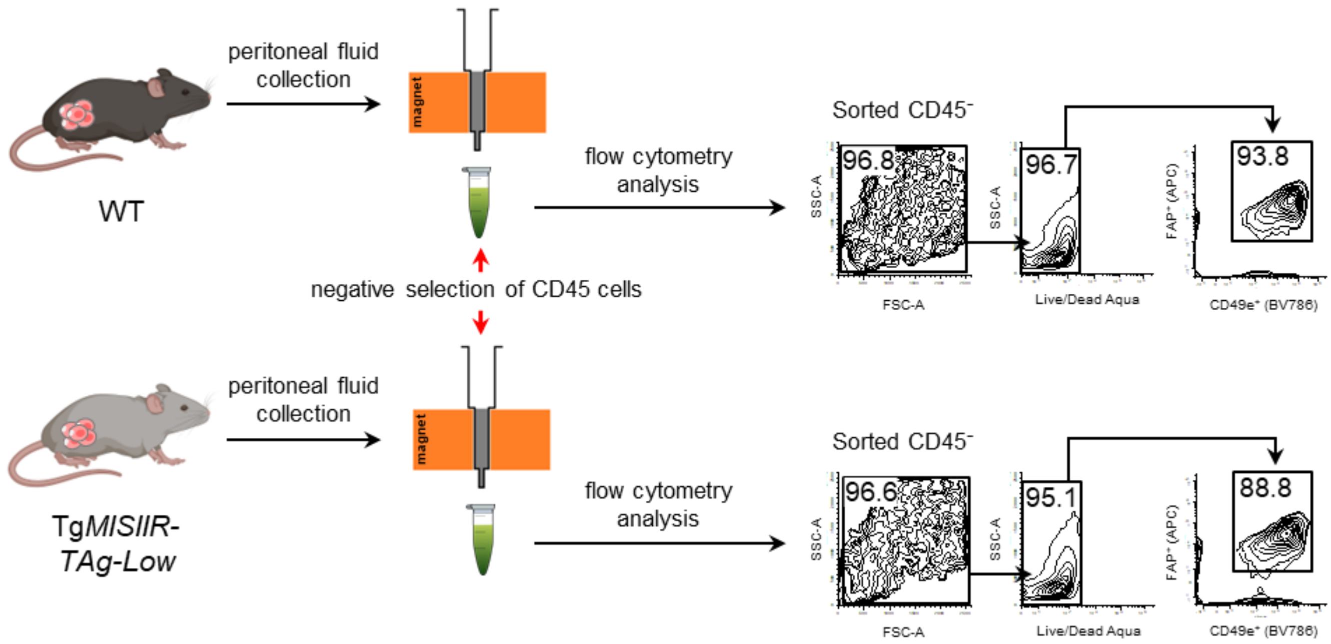

Figure S9. Graphical summary of CAFs isolation. $CD45^-$ cells were isolated from peritoneal fluids of MOVCAR 5009 tumors in control WT and Tg*MISIIR-TAg-Low* mice by negative selection using CD45 MicroBeads. The expression of CD49e and FAP antigens on the isolated CD45-negative cells was analyzed by flow cytometry.

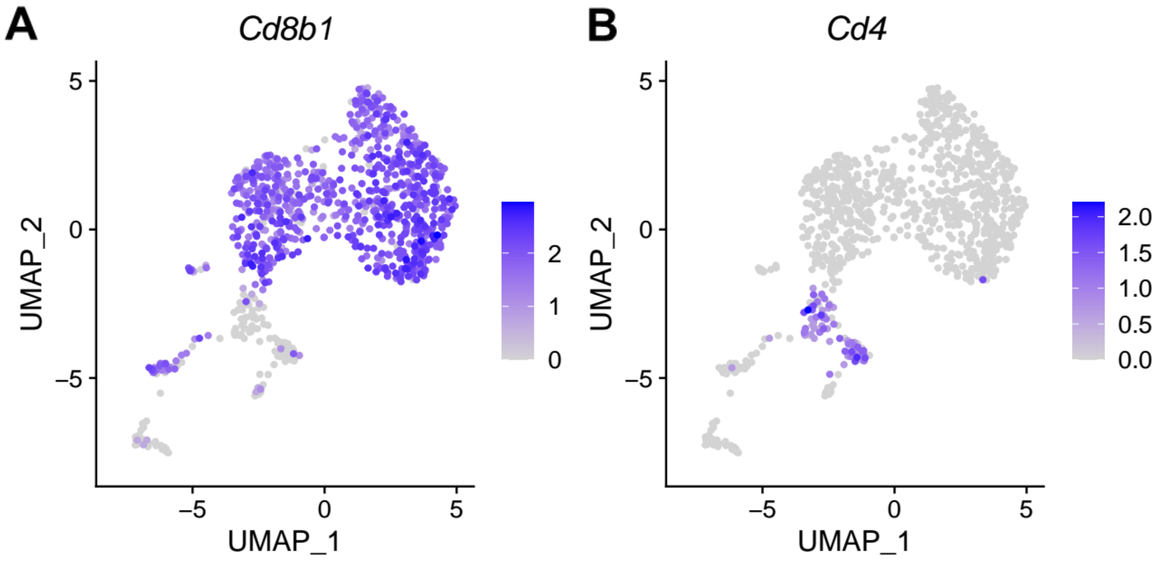

Figure S10. Transcriptional analysis of T cells isolated from the peritoneal TME of control and OV-CXCR4-A-treated tumors in WT and Tg*MISIIR-TAg-Low* mice. UMAP plots of re-clustered CD8^{+} T cells (**A**) and CD4^{+} T cells (**B**).

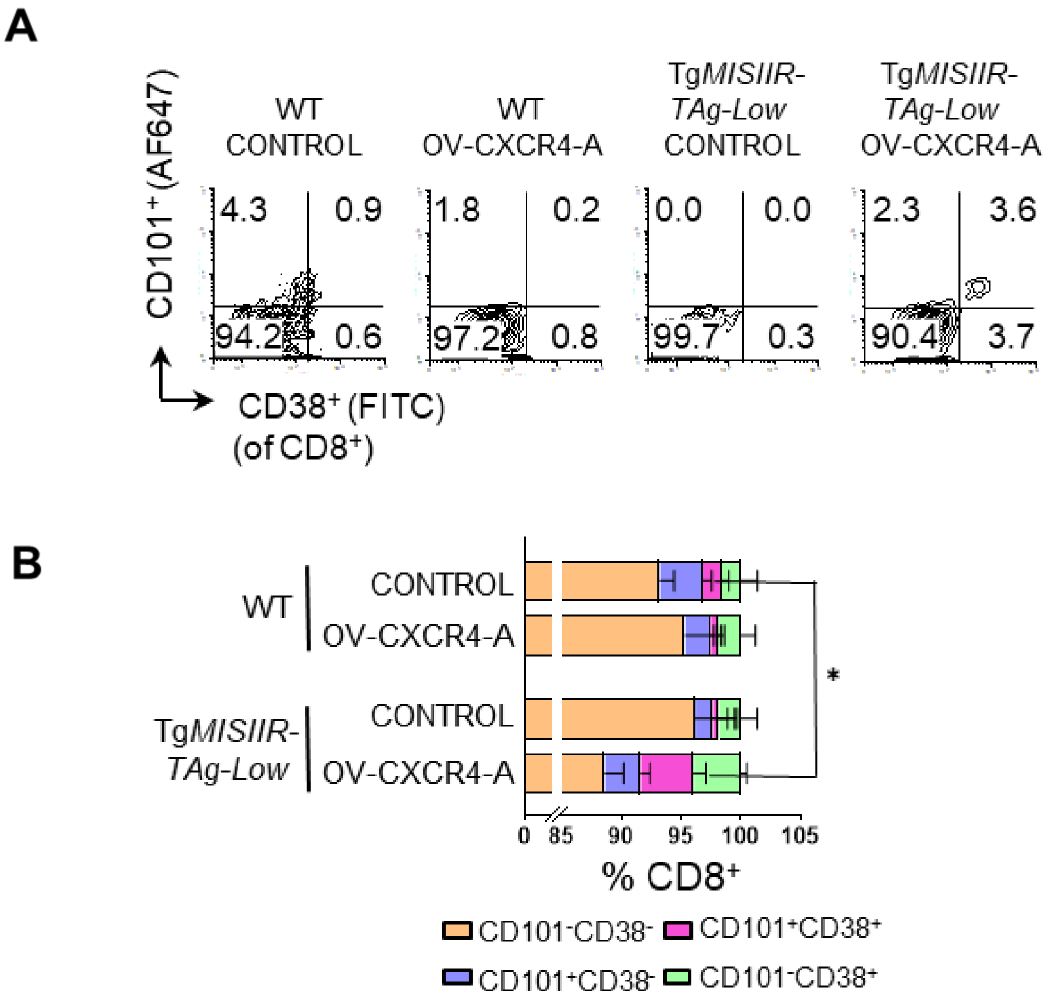

Figure S11. Expression of CD101 and CD38 antigens on CD8⁺ TALs in peritoneal cavities of tumor-bearing mice. (**A**) Representative flow cytometric plot of CD101 and CD38 expression on $CD8^+$ T lymphocytes in control and OV-CXCR4-A-treated tumors of WT and transgenic mice. Ten days after OV-CXCR4-A treatment, single-cell suspensions isolated from the peritoneal fluids of MOVCAR 5009 tumors were analyzed by flow cytometry for the percentage of CD101 and CD38 antigen expression among $CD8^+$ lymphocytes. (**B**) Graphical depiction of CD101 and CD38 expression by $CD8^+$ TALs. Data presented as mean ± SD. $^*p < 0.05$.

Table S1. Antibodies used in flow cytometry analysis.

Antibody	Clone	Catalog Number	Source
Extracellular staining			
CD8α-V450	53-6.7	560471	BD Biosciences
CD3-Alexa Fluor 700	17A2	561388	BD Biosciences
CD38-FITC	Ab90	558813	BD Biosciences
CD101-Ax647	307707	564473	BD Biosciences
CD45-PerCP-Cy5.5	30-F11	550994	BD Biosciences
CD45-V450	30-F11	560501	BD Biosciences
CD11b-BV786	M1/70	740861	BD Biosciences
Ly6G-PE	1A8	561104	BD Biosciences
Ly6C-FITC	AL-21	561085	BD Biosciences
I-A\I-E-BV605	M5/114.15.2	107639	BioLegend
F4/80-FITC	BM8	123107	BioLegend
CD25-FITC	3C7	101908	BioLegend
CD4-PE	GK1.5	553730	BD Biosciences
TCR_{Tag-I} tetramer-PE	-	TB-M539-1	MBL International
H2-K^b/TSYKFESV B8R tetramer-PE	-	-	Baylor College of Medicine, Houston
CD45-APC/Fire750	30-F11	103154	BioLegend
CD31-FITC	390	102405	BioLegend
CD90.2-PE/Cy7	30-H12	105325	BioLegend
CD49e-BV786	5H10-27	740863	BD Biosciences
PDPN-BV421	8.1.1	127423	BioLegend
FAP	73.3	MABC1145	Sigma-Aldrich
Mouse IgG1-APC	A85-1	560089	BD Biosciences
CD4-APC	RM4-4	116013	BioLegend
CD8α-FITC	CT-CD8α	MA5-17597	Thermo Fisher Scientific
Intracellular staining			
Foxp3-Alexa Fluor 647	MF23	560401	BD Biosciences
